# Direct Observation of Enhanced Iodine Binding within a Series of Functionalized Metal–Organic Frameworks with Exceptional Irradiation Stability

**DOI:** 10.1021/jacs.4c02405

**Published:** 2024-05-07

**Authors:** Jiangnan Li, Xinran Zhang, Mengtian Fan, Yinlin Chen, Yujie Ma, Gemma L. Smith, Iñigo J. Vitorica-yrezabal, Daniel Lee, Shaojun Xu, Martin Schröder, Sihai Yang

**Affiliations:** † Department of Chemistry, University of Manchester, Manchester, M13 9PL, U.K.; ‡ College of Chemistry and Molecular Engineering, Beijing National Laboratory for Molecular Sciences, Peking University, Beijing 100871, China; § Department of Chemical Engineering and Analytical Science, University of Manchester, Manchester M13 9PL, U.K.

## Abstract

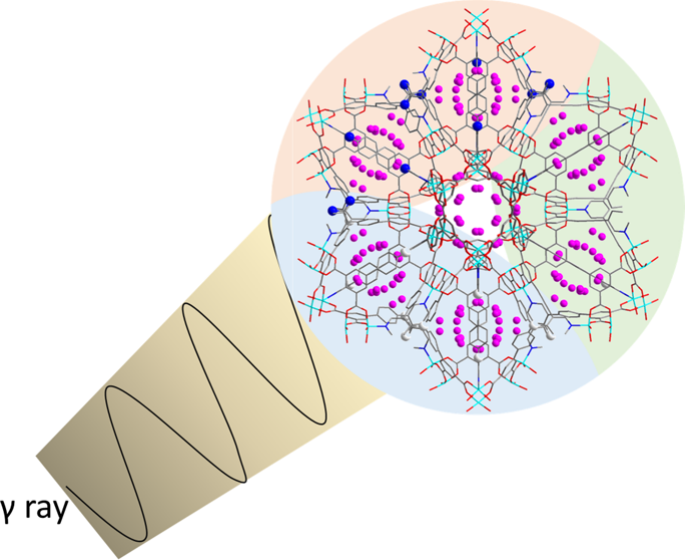

Optimization of active sites and stability under irradiation are important targets for sorbent materials that might be used for iodine (I_2_) storage. Herein, we report the direct observation of I_2_ binding in a series of Cu(II)-based isostructural metal–organic frameworks, MFM-170, MFM-172, MFM-174, NJU-Bai20, and NJU-Bai21, incorporating various functional groups (–H, −CH_3_, – NH_2_, –C≡C–, and −CONH–, respectively). MFM-170 shows a reversible uptake of 3.37 g g^–1^ and a high packing density of 4.41 g cm^–3^ for physiosorbed I_2_. The incorporation of −NH_2_ and –C≡C– moieties in MFM-174 and NJU-Bai20, respectively, enhances the binding of I_2_, affording uptakes of up to 3.91 g g^–1^. In addition, an exceptional I_2_ packing density of 4.83 g cm^–3^ is achieved in MFM-174, comparable to that of solid iodine (4.93 g cm^–3^). *In situ* crystallographic studies show the formation of a range of supramolecular and chemical interactions [I···N, I···H_2_N] and [I···C≡C, I–C=C–I] between −NH_2_, –C≡C– sites, respectively, and adsorbed I_2_ molecules. These observations have been confirmed via a combination of solid-state nuclear magnetic resonance, X-ray photoelectron, and Raman spectroscopies. Importantly, γ-irradiation confirmed the ultraresistance of MFM-170, MFM-174, and NJU-Bai20 suggesting their potential as efficient sorbents for cleanup of radioactive waste.

## Introduction

The global pledge to achieve Net Zero emissions over the coming decades places an urgent driver for clean energy.^1^ Nuclear fuel as a clean and efficient energy resource is receiving more attention than ever, accounting for ca. 14.5% of the UK’s electricity in 2020.^2^ However, radioactive wastes such as ^3^H, ^85^Kr, and ^129^I generated from the processing of nuclear fission have significant impacts on the environment. Isotopes of iodine, ^129^I and ^131^I, are common nuclear waste contaminants that can cause serious health problems.^3,4^ Therefore, the capture and storage of these isotopes are crucial. Also, I_2_ has been validated as an exceptional propellant with high energy efficiency for space missions and outperforming traditional propellants such as Xe.^5,6^ An optimal iodine storage medium is required in order to create compact, light engines that would make large networks of small satellites commercially viable. Thus, the development of iodine storage media is urgently needed to promote the abatement of environmental issues and to facilitate space exploration.

Solid sorbents such as zeolites,^7^ chalcogels,^8^ and organic polymers^9^ have been investigated for iodine adsorption. However, their limited porosity and/or lack of active sites have restricted the design of improved capture systems. In addition, the radioactivity of ^129^I and ^131^I can cause irreversible damage to the structure and porosity of the sorbent.^10−12^ The optimization of interactions between the sorbents and substrates is crucial to the discovery of new functional materials with high adsorption and irradiation stability.^13^ Metal–organic framework (MOF) materials, highlighted by their high crystallinity, are promising systems for the refinement of host–guest interactions, promoting their applications in gas storage,^14^ substrates separation,^15^ and cleanup of toxic wastes.^16^ Although a series of MOFs have been screened for I_2_ adsorption, such as I^–^-decorated Cu_4_I_4-_MOF (0.14 g g^–1^),^17^ -SH-functionalized MIL-53-SH(Al) (0.33 g g^–1^),^18^ and defect-containing UiO-66-FA (2.25 g g^–1^),^19^ the direct visualization of adsorbed I_2_ molecules within the pores has only been achieved in a limited few cases.^20−35^ Meanwhile, the stability of MOFs (as well as those loaded with I_2_) upon γ-irradiation has also been poorly explored to date.^10−13^

Herein, we report the direct observation of binding of I_2_ within a series of Cu(II)-based isostructural MOFs, namely MFM-170,^36^ MFM-172, MFM-174, NJU-Bai20,^37^ and NJU-Bai21,^38^ which incorporate −H, −CH_3_, –NH_2_, –C≡C, and −CONH– groups, respectively. This affords an excellent platform to investigate their effects on I_2_ binding. The loss of crystallinity and adsorption capacity of NJU-Bai21 suggest that −CONH– is intolerant to caustic I_2_. In contrast, MFM-170, MFM-172, MFM-174, and NJU-Bai20 exhibit highly reversible adsorption of I_2_.MFM-170 shows an uptake of 3.37 g g^–1^ and packing density of 4.11 g cm^–3^. The introduction of the −NH_2_ group in MFM-174 results in an enhanced uptake of 3.91 g g^–1^ with an exceptional I_2_ packing density of 4.83 g cm^–3^, which is close to that of solid iodine (4.93 g cm^–3^), representing the most efficient pristine MOF for I_2_ storage. The binding domains of I_2_ in these materials have been determined by synchrotron single crystal X-ray diffraction (SCXD). Active sites based upon –NH_2_ and –C≡C– can enhance the adsorption via strong host–guest interactions [I···N, I···H_2_N–] and [I···C≡C, I–C=C–I], respectively, representing the first example of direct observation of binding of I_2_ to these functional groups. Solid-state nuclear magnetic resonance (*ss*NMR) and X-ray photoelectron spectroscopy (XPS) further confirmed these host–guest binding interactions. Importantly, MFM-170, MFM-174, and NJU-Bai20 exhibit ultraresistance toward γ-irradiation up to 1750 kGy, demonstrating their promise for both capture and storage of radioactive iodine species.

## Results and Discussion

### Synthesis and Structural Analysis of MOFs

**Figure 1:**
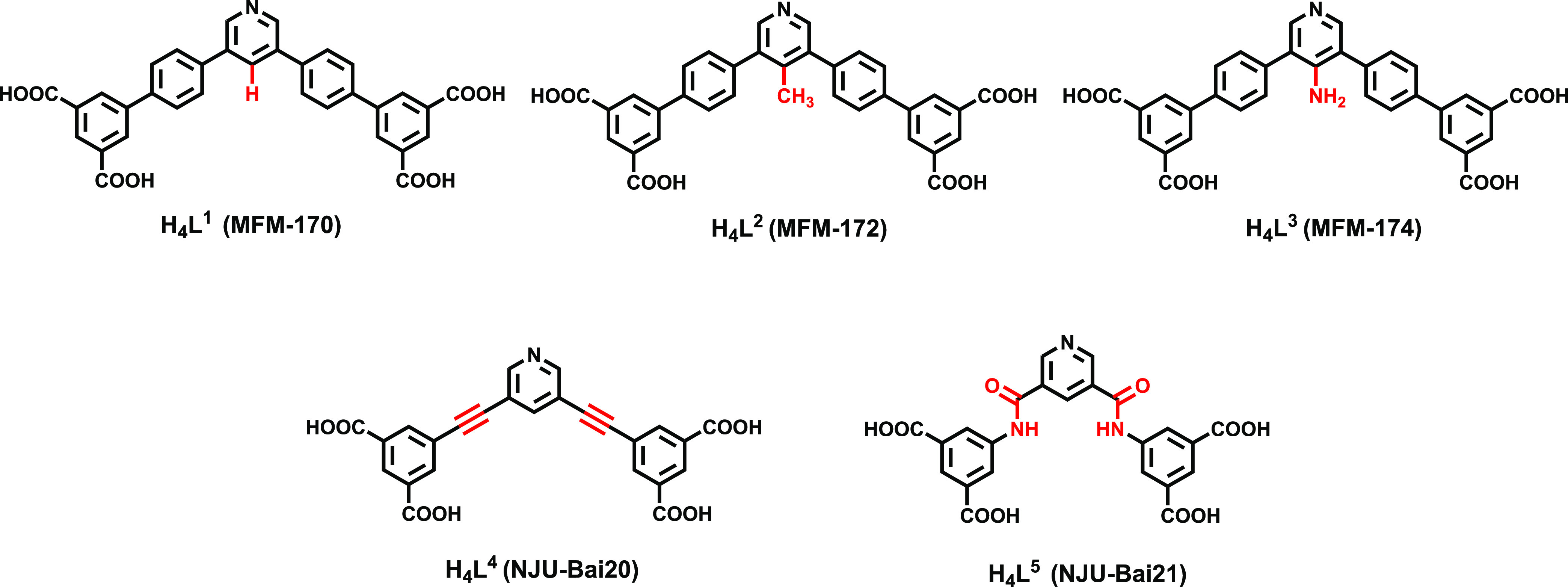
Chemical structure of ligands for the synthesis of target MOFs. Functional groups are highlighted in red.

**Figure 2:**
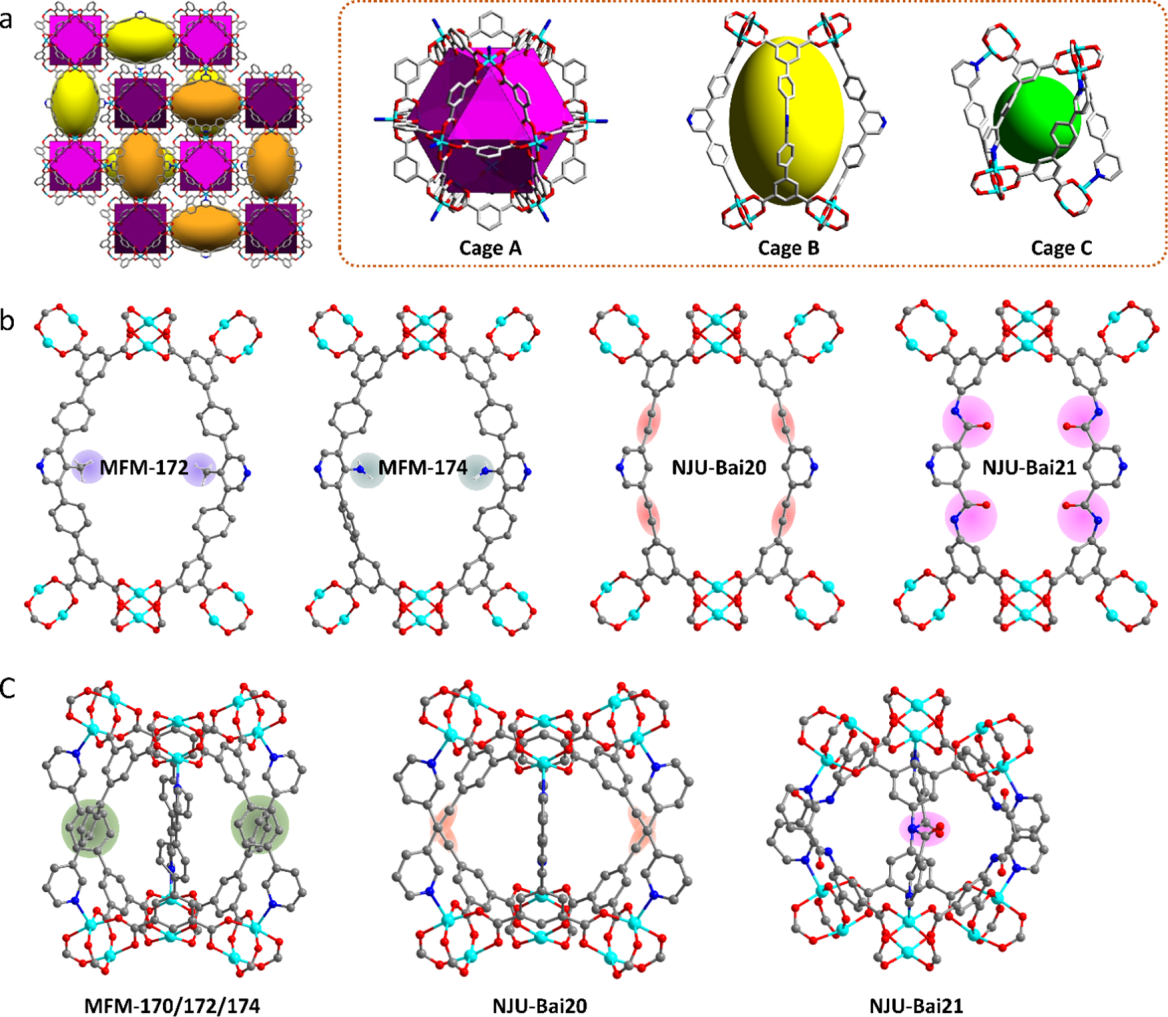
View of the crystal structures of MOFs. (a) The assembly of cages and the three types of cages in these isostructural MOFs, (MFM-170 is shown as an example); (b) cage C and binding sites shown in MFM-172, MFM-174, NJU-Bai20, and NJU-Bai21; (c) cage A and binding sites shown in MFM-170, MFM-172, MFM-174, NJU-Bai20, and NJU-Bai21 (C, grey; O, red; Cu, cyan; N, blue).

MFM-170, MFM-172, MFM-174, NJU-Bai20, and NJU-Bai21 were synthesized via solvothermal reactions of Cu(NO_3_)_2_·2.5H_2_O with H_4_L^1^, H_4_L^2^, H_4_L^3^, H_4_L^4^, and H_4_L^5^ (Figure 1), respectively, in dimethylformamide (DMF). Single crystal and powder X-ray diffractions confirm that these MOFs are isostructural and crystallize in the cubic space group *I*m-3̅m, affording a rare ***txt*** topology. Two Cu(II) ions are coordinated to four carboxylate groups to form a {Cu_2_} paddlewheel. Within each paddlewheel, one Cu(II) ion coordinates to the pyridyl N-donor from the linker at the axial site and the other binds to a water molecule. The latter affords an open Cu(II) site upon desolvation. The frameworks are assembled by the alternative packing of three types of cages (denoted as A, B, and C) (Figure 2a). The spherical cage A in all of the MOFs has an inner diameter of 13.1 Å. The oval-shaped cage B in MFM-170, MFM-172, MFM-174, NJU-Bai20, and NJU-Bai21 exhibits a diameter of 12.9 × 22.1, 9.9 × 22.1, 9.8 × 22.1, 9.7 × 19.9, and 9.0 × 19.2 Å, respectively, and these are decorated with –H, −CH_3_, –NH_2_, –C≡C–, and −CONH– groups, respectively. These point into the center of cage B and provide potential binding domains for substrate molecules (Figure 2b). Cage C, constructed by six linkers and six {Cu_2_} paddlewheels, exhibits the same pore environment in MFM-170, MFM-172, and MFM-174 with a diameter of 8.0 × 11.7 Å. Incorporation of –C≡C– and −CONH– groups in NJU-Bai20 and NJU-Bai21 affords dimensions of 9.8 × 9.6 and 8.7 × 7.2 Å, respectively (Figure 2c). Desolvated MFM-170, MFM-172, MFM-174, NJU-Bai20, and NJU-Bai21 show Brunauer–Emmett–Teller (BET) surface areas of 2408, 2076, 2251, 2081, and 1372 m^2^ g^–1^, respectively, (Table S2) derived from the adsorption isotherms.

### Studies of I_2_ Adsorption

**Figure 3:**
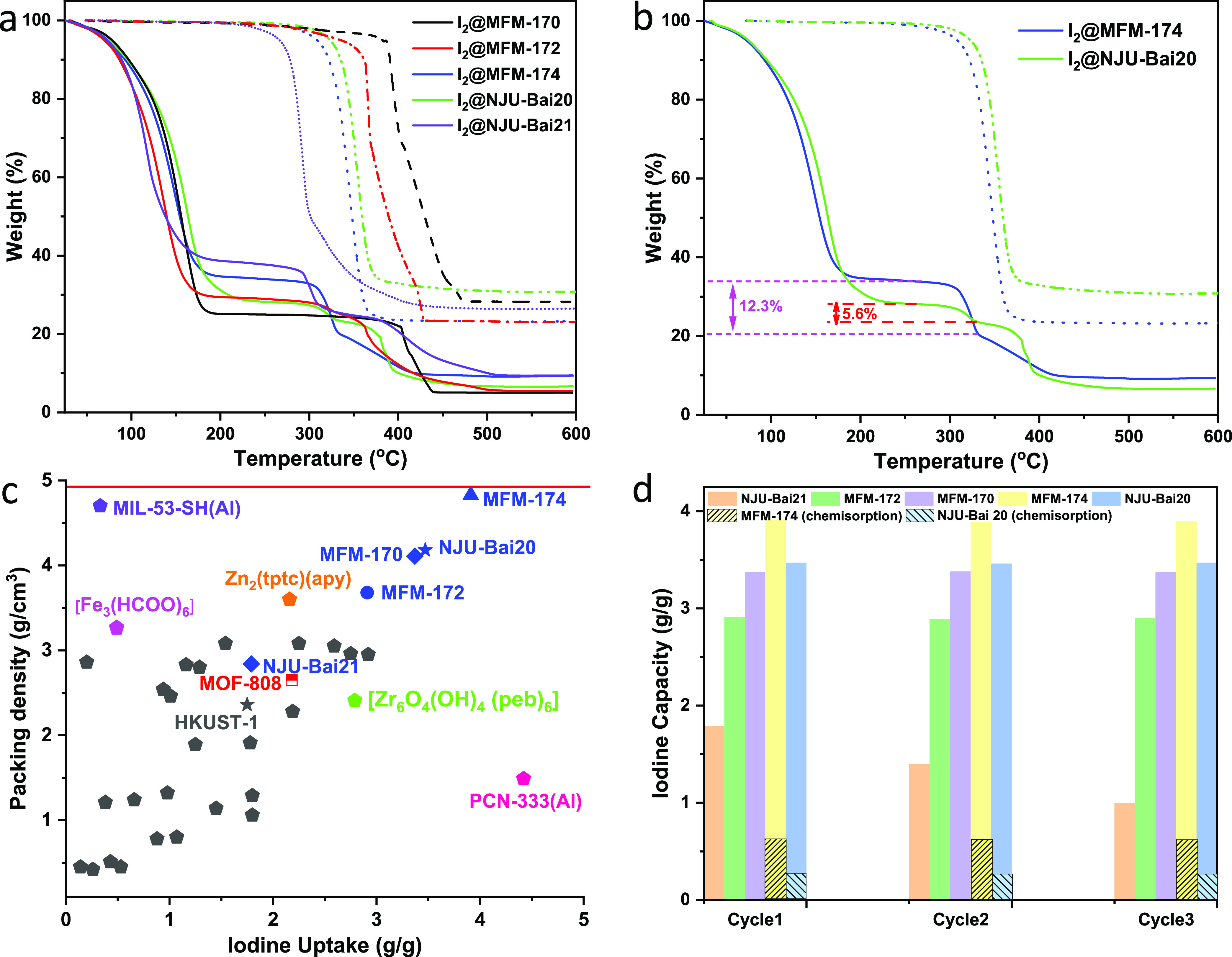
Iodine adsorption data in MOF materials. (a) TGA plots for activated and I_2_-loaded MOFs; (b) TGA plots for activated and I_2_-loaded MFM-174 and NJU-Bai20; (c) comparison of I_2_ uptakes and packing density of these MOFs and selected leading MOFs in literature (detailed data are summarized in Supporting Information Table S6; data for polymers, COFs, and activated carbons are given in Supporting Information Table S7); (d) cycling results for these MOFs (orange: NJU-Bai21; green: MFM-172; violet: MFM-170; yellow: MFM-174; teal: NJU-Bai20; shadow pattern indicates chemisorption percentage). The cycling tests were carried out without removing the chemisorbed component between each cycle.

I_2_ adsorption was investigated via diffusion of I_2_ vapor into desolvated MOF at 80 °C under N_2_ atmosphere followed by thermogravimetric analysis (TGA). The investigation of the thermal stability of bare MOFs demonstrates that MFM-170 possesses the highest stability up to 380 °C and MFM-172, MFM-174, NJU-Bai20, and NJU-Bai21 decompose at 350, 330, 325, and 290 °C, respectively (Figure 3a,b). Scanning electron microscopy (SEM) images confirm (i) the absence of morphological changes for the crystallites of these MOFs upon adsorption of I_2_ and (ii) the homogeneous distribution of I_2_ throughout the samples and the absence of bulk I_2_ on the surface (Figure S21). Uptakes of I_2_ were recorded as 1.79 (I_2_/Cu = 2.16), 2.91 (I_2_/Cu = 3.99), 3.37 (I_2_/Cu = 4.53), 3.47 (I_2_/Cu = 3.95), and 3.91 (I_2_/Cu = 5.37) g g^–1^ for NJU-Bai21, MFM-172, MFM-170, NJU-Bai20, and MFM-174, respectively (Figure 3a). These adsorption capacities compare favorably with state-of-the-art MOFs (Figure 3c), such as PCN-333(Al) (4.42 g g^–1^),^39^ {Zr_6_O_4_(OH)_4_(peb)_6_} (peb^2–^ = 4,4′-[1,4-phenylenebis(ethyne-2,1-diyl)]-dibenzoate) (2.79 g g^–1^),^40^ MOF-808 (2.18 g g^–1^),^41^ amino-functionalized MIL-125(Ti)-NH_2_ (1.7 g g^–1^),^42^ CAU-1(Al)-NH_2_ (1.3 g g^–1^),^42^ UiO-67-(NH_2_)_2_ (1.21 g g^–1^),^43^ TMU-16-NH_2_ (1.28 g g^–1^),^45^ MIL-101-NH_2_ (0.31 g g^–1^),^46^ and MIL-53-NH_2_ (0.18 g g^–1^).^47^ The high I_2_ adsorption capacity of MFM-174 also results in an exceptional packing density of 4.83 g cm^–3^, which is comparable with that for solid iodine (4.93 g cm^–3^), representing the most efficient MOF for I_2_ storage (Figure 3c). Recent studies on a series of metallocyclic cage complexes incorporating dipalladium corners show I_2_ uptakes of up to 0.83 g g^–1^.^48^

Compared with MFM-170, a 13.6% reduction in I_2_ uptake is observed for the methyl-decorated MFM-172, but a 16% enhancement is observed for the amine-decorated MFM-174, although both MOFs experience a reduction in the BET surface area (13 and 6.5%, respectively) due to the incorporation of functional groups. This indicates that −CH_3_ groups provide limited binding but block the access of I_2_ molecules. In contrast, −NH_2_ groups provide enhanced binding to I_2_. Although NJU-Bai20 possesses a surface area similar to that of MFM-172 (2081 and 2076 m^2^ g^–1^, respectively), NJU-Bai20 exhibits a 19% enhancement in I_2_ uptake, indicating that –C≡C– groups can boost I_2_ adsorption. It is worth noting that a two-step weight loss is observed for I_2_-loaded MFM-174 and NJU-Bai20 before the onset of framework decomposition (Figure 3b), and the second weight loss at 300 °C is ascribed to chemisorbed I_2_ in MFM-174 and NJU-Bai20 (0.61 and 0.25 g g^–1^, respectively). Inductively coupled plasma atomic emission spectroscopy (ICP-AES) was applied to confirm the chemisorption of I_2_ in MFM-174 and NJU-Bai20. Residual iodine, 38 and 17%, was observed for samples of I_2_-loaded MFM-174 and NJU-Bai20, respectively, calcined at 300 °C under air. This corresponds to 0.56 and 0.28 g g^–1^ of chemisorbed I_2_ and is entirely consistent with TGA results (Table S5). Studies of adsorption–desorption of I_2_ in NJU-Bai20, MFM-170, MFM-172, and MFM-174 over 3 cycles confirmed only minor changes in the adsorption capacity and crystallinity of samples (Figures 3d and S16–S20) whereas NJU-Bai21 lost ∼40% adsorption capacity, accompanied by a reduction in crystallinity, indicating poor tolerance of the amide group toward caustic I_2_.

### Determination of Binding Domains

**Figure 4:**
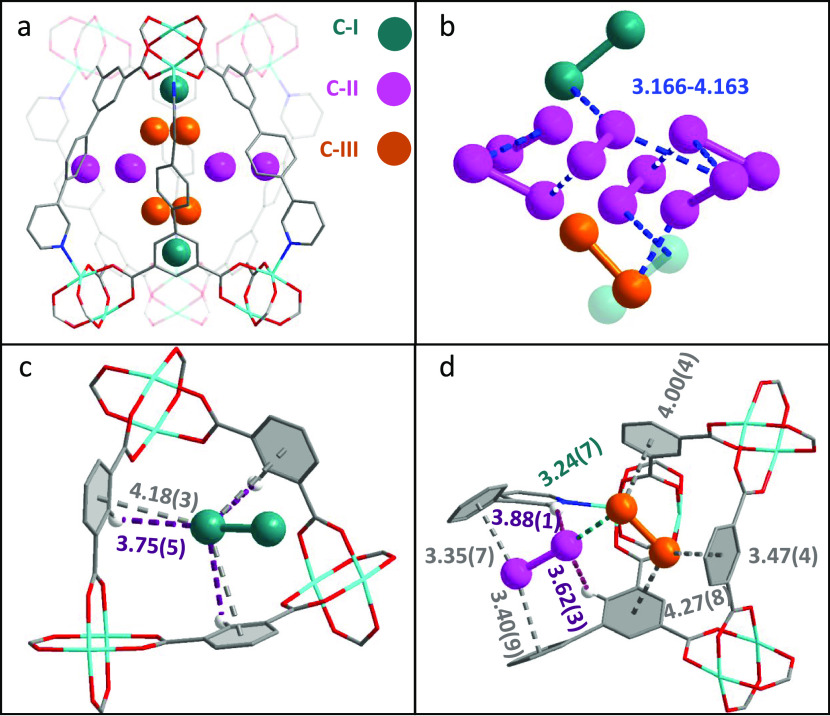
Views of crystal structures of I_2_-loaded MFM-170. Views of (a) I_2_ binding sites in MFM-170, (b) packing of I_2_ molecules in cage C of MFM-170, (c) intermolecular interactions between I_2_^C–I^ and MFM-170, (d) intermolecular interactions between I_2_^C–II^, I_2_^C–III^, and MFM-170 (C, grey; O, red; Cu, cyan; N, blue; H, white; all units are in Å).

Synchrotron SCXD of I_2_-loaded MFM-170, MFM-172, MFM-174, and NJU-Bai20 yielded crystal structures of [Cu_2_(C_33_H_17_NO_8_)·(H_2_O)_0.79_·4.39I_2_], [Cu_2_(C_34_H_19_NO_8_)·(H_2_O)·1.60I_2_], [Cu_2_(C_33_H_18_N_2_O_8_)· (H_2_O)·1.84I_2_], and [Cu_2_(C_25_H_9_N_2_O_8_)·(H_2_O)_0.96_·1.82I_2_], respectively (Tables S3 and S4). It should be noted that disordered I_2_ molecules in the pores were not considered. Only moderate amounts of I_2_ were loaded into the MOFs to retain optimal crystallinity of the resultant materials. A total of ten binding sites are observed in I_2_-loaded MFM-170. Three binding sites (denoted as I_2_^C–I^, I_2_^C–II^, and I_2_^C–III^) are located in cage C (Figure 4a and Table S4). I_2_^C–I^ (I_2_/{Cu_2_} = 0.341) sits approximately coplanar with the three phenyl rings that link the {Cu_2_} paddlewheels and is stabilized by [I···phenyl = 4.18(3) Å] and [I···H–C (phenyl) = 3.75(5) Å] interactions (each appear 3 times, Figure 4c). Site I_2_^C–III^ is toward the center of the cage, occupying a similar location with I_2_^C–I^ but showing a lower occupancy (I_2_/{Cu_2_} = 0.292). I_2_^C–II^ (I_2_/{Cu_2_} = 0.468) is nearly coplanar with the phenyl rings that directly connect the pyridine ring and is sandwiched by two adjacent phenyl rings. In addition to the interaction with phenyl rings [I···phenyl = 3.47(4), 4.00(4), and 4.27(8) Å], I_2_^C–III^ is further stabilized by halogen bonding [I_2_^C–III^···I_2_^C–II^ = 3.24(7) Å] (Figure 4d). Similar binding sites and host–guest interactions are also observed in I_2_-loaded MFM-172 with occupancies of I_2_^C–I^/{Cu_2_}, I_2_^C–II^/{Cu_2_}, and I_2_^C–III^/{Cu_2_} being 0.233, 0.215, and 0.096, respectively (Table S4 and Figure S1). Two binding sites similar to I_2_^C–I^ and I_2_^C–II^ in MFM-170 are observed in I_2_-loaded MFM-174 with occupancies of 0.126 and 0.083, respectively, and in NJU-Bai20 with occupancies of 0.175 and 0.135, respectively (Table S4 and Figures S2–S3). The high occupancy of I_2_ in cage C of MFM-170 results in compression of I_2_ molecules toward the center, contributing to the high packing density of I_2_ (Figures 4b and S4).

**Figure 5:**
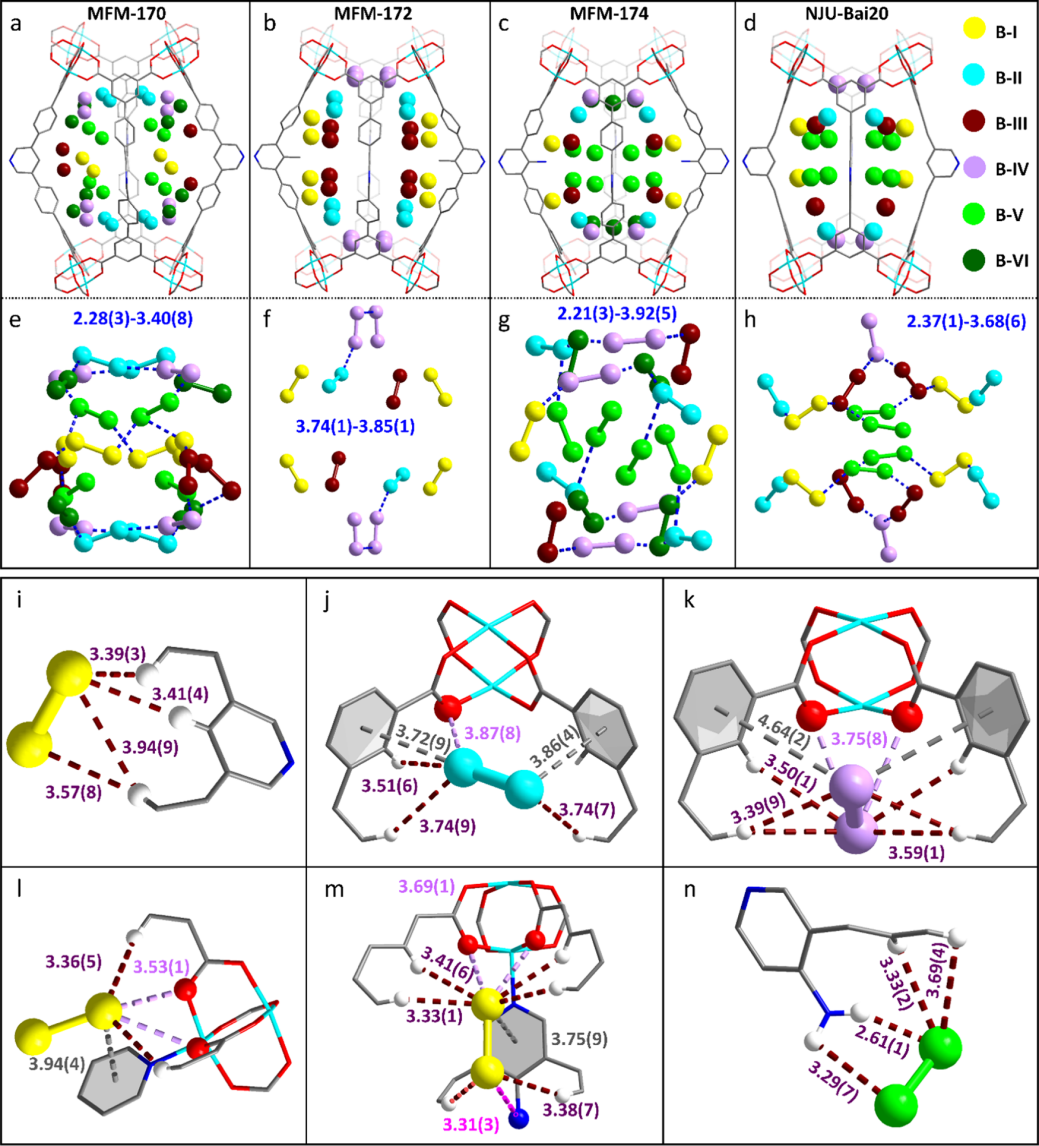
Views of crystal structures of I_2_-loaded MFM-170, MFM-172, −174, and NJU-Bai20. Views of I_2_ binding sites in cage B of (a) MFM-170, (b) MFM-172, (**c**) MFM-174, and (d) NJU-Bai20 and of packing of I_2_ in cage B of (e) MFM-170, (f) MFM-172, (g), MFM-174, and (h) NJU-Bai20. Views of intermolecular interactions between (i) I_2_^B–I^ and MFM-170, (j) I_2_^B–II^ and MFM-170, (k) I_2_^B–IV^ and MFM-170, (**l**) I_2_^B–I^ and MFM-172, (m) I_2_^B–I^ and MFM-174, and (n) I_2_^B–V^ and MFM-174 (C, grey; O, red; Cu, cyan; N, blue; H, white; all units are in Å).

Another six binding sites (denoted as I_2_^B–I^, I_2_^B–II^, I_2_^B–III^, I_2_^B–IV^, I_2_^B–V^, and I_2_^B–VI^) are observed in cage B of I_2_-loaded MFM-170 (Figure 5a and Table S4). The high occupancy of I_2_ in cage B facilitates the formation of multiple halogen bonds between adsorbed I_2_ molecules, compressing the packing of I_2_ to the center and stabilizing storage (Figure 5e). The I_2_ molecules in cage B are stabilized by hydrogen bonding or electrostatic interactions with hydrogen centers on phenyl/pyridyl rings (Figures 5i and S5). For example, I_2_^B–I^ (I_2_^B–I^ /{Cu_2_} = 0.783) is stabilized by 4-fold hydrogen bonding [I···H–C (pyridine) = 3.41(4) Å, I···H–C (phenyl) = 3.39(3), 3.57(8), and 3.94(9) Å] (Figure 5i). Similar supramolecular interactions are found for I_2_^B–III^ (I_2_^B–III^ /{Cu_2_} = 0.414), I_2_^B–V^ (I_2_^B–V^ /{Cu_2_} = 0.378), and I_2_^B–VI^ (I_2_^B–VI^ /{Cu_2_} = 0.365) (Figure S5). I_2_^B–II^ (I_2_^B–II^ /{Cu_2_} = 0.578) and I_2_^B–IV^ (I_2_^B–IV^ /{Cu_2_} = 0.409) sit approximately coplanar with the two phenyl rings that link the {Cu_2_} paddlewheels, and they are further stabilized by [I···O = 3.87(8) Å, I···phenyl = 3.72(9), and 3.86(4) Å, I···H–C (phenyl) = 3.51(6), 3.74(7), and 3.74(9) Å] and [I···O = 3.75(8) Å, I···phenyl = 4.64(2), I···H–C (phenyl) = 3.39(9), 3.50(1), and 3.59(1) Å (each appears twice)] interactions, respectively (Figure 5j,k). Similar locations for I_2_^B–I^, I_2_^B–II^, I_2_^B–III^ and I_2_^B–IV^ are identified in cage B of I_2_-loaded MFM-172 (Figures 5b, S6 and Table S4). However, due to the steric hindrance caused by −CH_3_ groups, the distribution of I_2_ molecules tends toward the edge of the pore and is stabilized by multiple dipole–dipole interactions (Figures 5f and S6). The I_2_ packing in MFM-172 is thus less efficient than that in MFM-170 (Figure 5e). Interestingly, in MFM-174 the −NH_2_ groups provide strong binding sites to I_2_^B–I^ (I_2_/{Cu_2_} = 0.518) via [I···N = 3.31(3) Å], [I···O = 3.691(3) and 3.69(1) Å] and [I···H–C (phenyl) = 3.33(1), 3.33(1), 3.41(6), 3.41(6), 3.38(7) and 3.38(7) Å] interactions, and to I_2_^B–V^ (I_2_/{Cu_2_} = 0.136) via [I···H_2_N = 2.61(1) and 3.29(7) Å; I···H–C (phenyl) = 3.33(2) and 3.69(4) Å] contacts (Figure 5m,n). This strong supramolecular binding promotes the formation of halogen bonding [2.21(3)–3.92(5) Å] between adsorbed I_2_ molecules, and this facilitates the efficient packing of I_2_ molecules in cage B, consistent with the adsorption study (Figures 5g, S7 and Table S4). Similar locations for I_2_^B–I^, I_2_^B–II^, I_2_^B–III^, I_2_^B–IV^, and I_2_^B–V^ and host–guest interactions are identified in cage B of I_2_-loaded NJU-Bai20 (Figures 5d, S8 and Table S4), except that the I···phenyl interactions are replaced by I···C≡C. It is worth noting that I_2_^B–V^ also promotes halogen bonding [2.37(1)–3.68(6) Å] and boosts the I_2_ uptake in NJU-Bai20 (Figure 5h).

Three binding sites (denoted as I_2_^A-I^, I_2_^A-II^, and I_2_^A-III^) are located in cage A of NJU-Bai20 (Figure S10a). I_2_^A-I^ (I_2_/{Cu_2_} = 0.307), I_2_^A-II^ (I_2_/{Cu_2_} = 0.149), and I_2_^A-III^ (I_2_/{Cu_2_} = 0.104) are stabilized by multiple [I···O_2_H = 3.42(3); I···O = 3.91(4) Å], [I···O = 4.32(1) Å], and [I···O_2_H = 2.89(8) Å] interactions, respectively (Figure S10b–d). Meanwhile, a similar binding site I_2_^A-I^ and host–guest interactions are observed in I_2_-loaded MFM-170 (Figure S9). These multiple weak supramolecular interactions work together to afford efficient physisorption of I_2_ in MFM-170. Similar binding in cage A is expected for the other MOFs, but could not be fully modeled owing to the presence of severe structural disorder in these cages. However, the direct visualization of host–guest interactions has revealed the key role of –NH_2_ and –C≡C– sites in facilitating adsorption of I_2_. Although –NH_2_ and –C≡C– sites have been previously reported to enhance the adsorption of I_2,_^9,40,42−45^ to the best of our knowledge, this represents the first example of crystallographic observation of I_2_ binding to these active sites.

### Spectroscopic Studies

**Figure 6:**
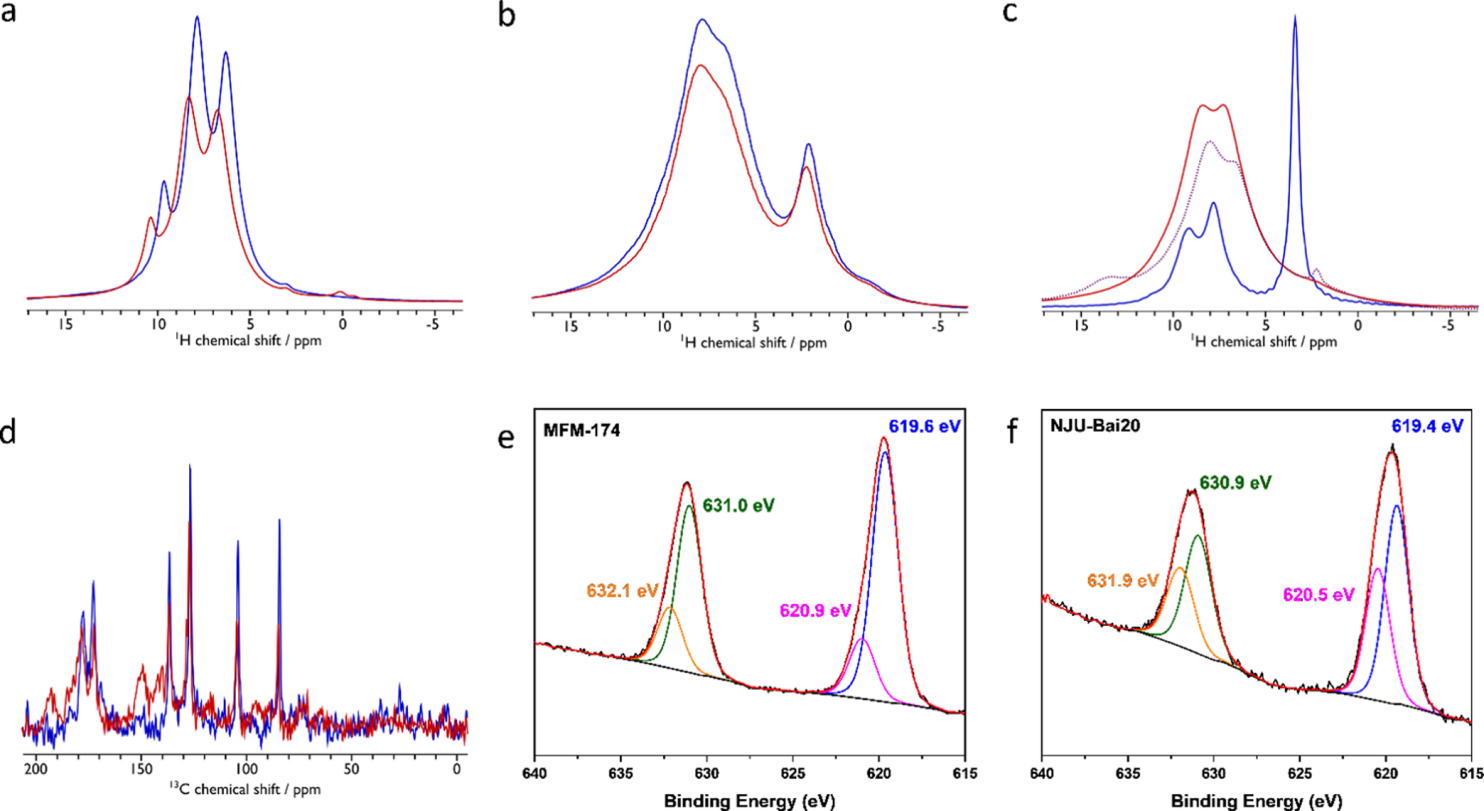
Spectroscopic data. ^1^H MAS NMR spectra of (a) bare MFM-170 (blue) and I_2_-loaded MFM-170 (red), (b) bare MFM-172 (blue) and I_2_-loaded MFM-172 (red), (c) bare MFM-174 (blue), I_2_-loaded MFM-174 (red), and regenerated MFM-174 (purple dash). (d) {^1^H-}^13^C CPMAS NMR spectra of bare NJU-Bai20 (blue) and I_2_-loaded NJU-Bai20 (red); (e) XPS spectra of I_2_-loaded MFM-174; (f) XPS spectra of I_2_-loaded NJU-Bai20.

Solid-state (ss)NMR spectroscopy was applied to investigate further the host–guest interactions. Upon adsorption of I_2_ in MFM-170, the peaks assigned to aromatic protons shift by ca. 1 ppm to lower field, suggesting the binding of I_2_ to the aromatic rings (Figure 6a); the corresponding ^13^C NMR spectra likewise show a shift of ca. 5 ppm for aromatic carbons (Figure S23). For I_2_-loaded MFM-172, no shifts of ^1^H peaks are observed for aromatic protons compared with the bare MOF (Figure 6b), indicating hindered interactions between I_2_ and aromatic rings owing to the presence of the −CH_3_ groups. The ^13^C resonances of aromatic carbons also show minimal changes in I_2_-loaded MFM-172 (Figure S24), consistent with the adsorption and crystallographic studies. A decrease in the intensity of the ^1^H NMR peak at 3.2 ppm associated with the –NH_2_ group in MFM-174 confirms the direct binding of I_2_ molecules to the –NH_2_ group (Figure 6c). Upon desorption by vacuum alone, the narrow ^1^H peak of –NH_2_ (3.2 ppm) cannot be restored, indicating the presence of residual I_2_ on this site, entirely consistent with the presence of chemisorption. Interestingly, upon adsorption of I_2_ in NJU-Bai20, intereaction with the –C≡C– triple bond is evidenced by the ^13^C NMR peak at 85 ppm decreasing in intensity, and the ^13^C resonance at 104 ppm, assigned to the aromatic carbon center next to the triple bond, moves to 128 ppm (Figure 6d). New peaks are observed in the ^13^C NMR spectrum of I_2_-loaded NJU-Bai20 at 141, 150, 183, and 193 ppm, and their broadness suggests the formation of [I–C=C–I] species. XPS was used to investigate the adsorbed I_2_ species in these MOFs. The XPS spectrum of I_2_-loaded MFM-170 and MFM-172 display two peaks at ∼632.2 and ∼620.7 eV, which are assigned to the I 3d_3/2_ and I 3d_5/2_ orbitals of the I_2_ molecule, respectively, indicating the presence of molecular I_2_ (Figures S27 and S28). In contrast, additional peaks are observed in I_2_-loaded MFM-174 (632.1 and 620.9 eV) and NJU-Bai20 (631.9 and 620.5 eV) (Figure 6e,f), confirming the presence of chemically sorbed iodine species in the two systems. Comparison of the Raman spectra of bare and I_2_-loaded MOFs exhibits blue shifts (Δ = 26 cm^–1^) of the characteristic I–I vibration at 180 cm^–1^ in solid I_2_, confirming strong host–guest interactions (Figures S32–S34). Additionally, the Raman spectra of I_2_-loaded MFM-174 and NJU-Bai-20 also display a broad peak at 173 cm^–1^, indicating the presence of additional adsorbed I_2_ species and strong interactions between adsorbed I_2_ and the framework,^43,44^ consistent with the observed additional XPS peaks for these systems.

### Studies of the Stability Upon γ-Irradiation

**Figure 7:**
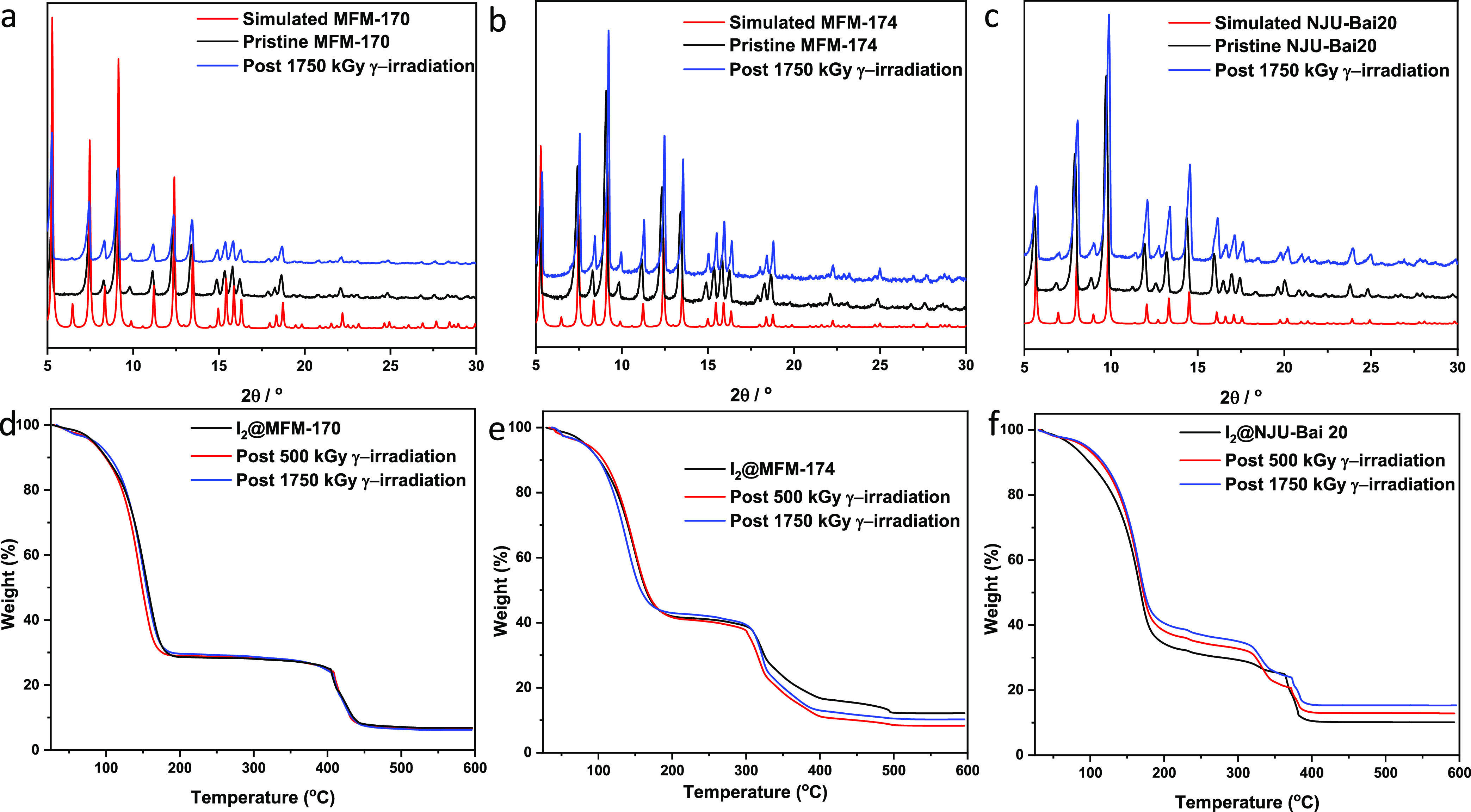
PXRD patterns of simulated, pristine, and post γ-irradiation MOFs. (a) MFM-170; (b) MFM-174; and (c) NJU-Bai20. Comparison of TGA results of fresh I_2_-loaded MOFs and post γ-irradiation (d) MFM-170, (e) MFM-174, and (f) NJU-Bai20.

γ-Radiation (^60^Co source) from 0 to 1750 kGy was applied to bare and I_2_-loaded MFM-170, MFM-174, and NJU-Bai20 to investigate their resistance toward irradiation and the effects of irradiation on the I_2_ uptake capacity. Powder X-ray diffraction (PXRD), SEM, infrared (IR) spectroscopy, and N_2_ adsorption isotherms were used to monitor the change in their structures upon γ-irradiation. Importantly, no new, unknown Bragg peak or reduction of N_2_ adsorption uptake was observed even after irradiation at 1750 kGy, confirming the stability of these MOFs under these conditions (Figures 7a–c, S29–S31, and S35). Although TOF-16,^49^ UiO-66-NH_2,_^50^ NU-1000^12^, and SIFSIX-3-Cu^11^ have been reported to be stable upon γ irradiation, the adsorption capacity of I_2_ post γ-irradiation in porous materials has not been investigated. Importantly, the TGA curves of samples post γ irradiation coincide with those of the fresh samples, demonstrating full retention of adsorbed I_2_ upon irradiation (Figure 7d,e). In contrast, the I_2_-loaded NJU-Bai20 displays a 5% enhancement of chemisorbed I_2_ after γ-irradiation, but the total uptake is unchanged, which is promising for the long-term storage of radioactive iodine (Figure 7f). This study demonstrates that MFM-170, MFM-174, and NJU-Bai20 are promising materials for the storage of nuclear wastes.

## Conclusions

The exploration of sorbent materials for the uptake of radioactive I_2_ is essential for the sustainable development of clean energy. Understanding host–guest binding at a molecular level provides key insights for the design of new materials. To date, only limited success has been achieved due to the severe disorder of adsorbed I_2_ molecules and uncertainty in the radiation stability of sorbent materials. The exceptional adsorption and packing density of I_2_ in a series of robust MOFs with various functional groups (e.g., −H, –NH_2_ and –C≡C−) as well as their high stability upon γ-irradiation have been established. The resistance of MFM-170, MFM-174, and NJU-Bai20 to γ-radiation confirms their potential for remediation of nuclear waste. Analysis of the I_2_-loaded MOFs by synchrotron X-ray single crystal diffraction and spectroscopic studies unravel the molecular details of the host–guest binding, with –NH_2_ and –C≡C– groups playing a key role in stabilizing I_2_ molecules in the pore. This study will promote the future design of efficient and stable sorbent materials for remediation of nuclear waste.

## Supporting Information Available

Additional experimental and structural details and methods; together with adsorption isotherms, BET, PXRD, EDX, and XPS data; and ssNMR, IR, and Raman spectra (PDF)
