# Supplementary material for: Direct Observation of Enhanced Iodine Binding within a Series of Functionalized Metal–Organic Frameworks with Exceptional Irradiation Stability
Source: J Am Chem Soc. 2024 May 7;146(20):14048–57. doi: 10.1021/jacs.4c02405 (PMC11117185; doi:10.1021/jacs.4c02405)
Supplement: Supplementary file 1 — ja4c02405_si_001.pdf [file ja4c02405_si_001.pdf]

Supplementary Information

Direct observation of enhanced iodine binding within a series of functionalised metal-organic frameworks with exceptional irradiation stability

Jiangnan Li[1,2], Xinran Zhang[1], Mengtian Fan[1], Yinlin Chen[1], Yujie Ma[1], Gemma L. Smith[1], Inigo Vitorica-yrezabal[1], Daniel Lee[3], Shaojun Xu[3], Martin Schröder[1]* and Sihai Yang[1,2]*

1. Department of Chemistry, University of Manchester, Manchester, M13 9PL, UK

M.Schroder@manchester.ac.uk; Sihai.Yang@manchester.ac.uk

2. College of Chemistry and Molecular Engineering, Beijing National Laboratory for Molecular Sciences, Peking University, Beijing 100871, China

Sihai.Yang@pku.edu.cn

3. Department of Chemical Engineering and Analytical Science, University of Manchester, Manchester M13 9PL, UK

1. Experimental section……3

1.1 Synthesis of MOF materials……3

1.2 BET measurements……4

1.3 Powder X-ray diffraction……4

1.4 SEM-EDX experiments……4

1.5 Iodine adsorption……4

1.6 Thermogravimetric analysis……4

1.7 X-ray photoelectron spectroscopy (XPS)……5

1.8 Solid-state nuclear magnetic resonance (*ss*NMR)……5

1.9 Synchrotron X-ray single crystal diffraction……5

1.10 γ-Irradiation……6

1.11 Raman spectroscopy……7

2. Data and views of crystal structures……8

3. Analysis of I_2 adsorption……19

4. PXRD patterns……22

5. SEM-EDX images……25

6. ssNMR spectra……27

7. X-ray photoelectron spectra (XPS)……29

8. Infrared spectra (IR)……30

9. Raman spectra……32

10. N_2 adsorption isotherms……34

11. References……35

1. Experimental section

1.1 Synthesis of MOF materials

All materials were purchased from commercially available sources and used without further purification. Iodine (99.8% ACS) was purchased from Sigma-Aldrich. The synthesis of MFM-170, NJU-Bai20 and NJU-Bai21 were synthesised by using the reported methods[1-3].

Synthesis of H_4L^2 and H_4L^3

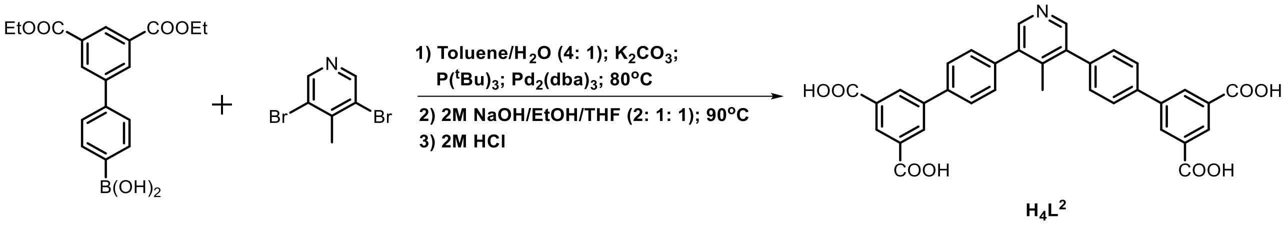

3',5'-Bis(ethoxycarbonyl)-[1,1'-biphenyl]-4-yl)boronic acid (1.5 g, 4.4 mmol), 3, 5-dibromo-4-methylpyridine (0.41 g, 1.8 mmol) and K_2CO_3 (0.73 g, 5.4 mmol) were dissolved in a solution of toluene and water (125 mL, 4:1 v/v) and degassed at 60 °C for 1 h. Tritertbutylphosphine (1 M solution in toluene, 0.1 mL, 0.1 mmol) and $Pd_2(dba)_3$ (82 mg, 0.1 mmol) were added and the reaction heated at 80°C for overnight. The product was extracted into CH_2Cl_2 and the solvent removed under vacuum before being recrystallized from CH_2Cl_2 with EtOH to give off-white powder. The powder was suspended in a solution of sodium hydroxide (2 M, 50 mL), THF (25 mL) and EtOH (25 mL) and stirred at 90°C for overnight. The organic solvent was removed and the aqueous solution acidified with HCl (2 M) until a white solid precipitated. After filtration, the solid powder was obtained (0.84 g, 89 %). ^{1}H NMR (300 MHz, DMSO-d_6) δ ppm: 8.49 (2 H, s), 8.48 (2 H, s), 8.46 (4 H, s), 7.9 (4 H, d, J = 8.2 Hz), 7.65 (4 H, d, J = 8.2 Hz). ^{13}C NMR (100 MHz, DMSO-d_6): 166.44, 151.07, 141.84, 140.31, 139.17, 138.69, 135.38, 132.26, 131.39, 130.49, 129.14, 127.31, 19.36.

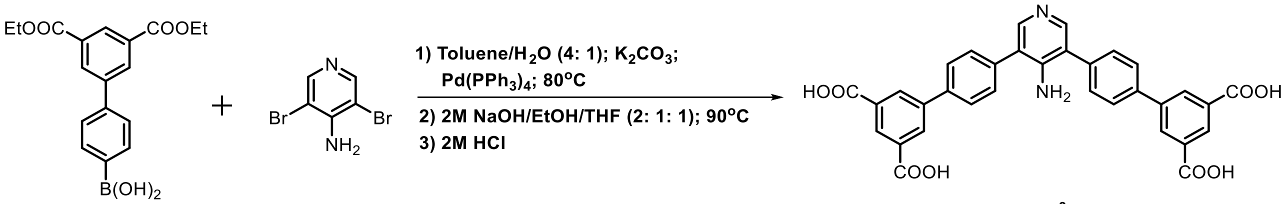

3',5'-Bis(ethoxycarbonyl)-[1,1'-biphenyl]-4-yl)boronic acid (1.5 g, 4.4 mmol), 3,5-dibromopyridine-4-amine (0.41 g, 1.8 mmol) and K_2CO_3 (0.73 g, 5.4 mmol) were dissolved in a solution of toluene and water (125 mL, 4:1 v/v) and degassed at 60°C for 1 h. $Pd(PPh_3)_4$ (89 mg, 0.1 mmol) were added and the reaction heated at 80°C for overnight. The product was extracted into CH_2Cl_2 and the solvent removed under vacuum before being recrystallized from CH_2Cl_2 with EtOH to give off-white powder. The powder was suspended in a solution of NaOH (2 M, 50 mL), THF (25 mL) and EtOH (25 mL) and stirred at 90°C for overnight. The organic solvent was removed and the aqueous solution acidified with HCl (2 M) until a white solid precipitated. After filtration the solid powder was obtained (0.81 g, 88 %). ^{1}H NMR (500 MHz, DMSO-d_6) δ ppm 8.55 (s, 2H), 8.52 (d, J = 1.5 Hz, 4H), 8.17 (s, 2H), 7.98 (d, J = 7.9 Hz, 4H), 7.73 (d, J = 7.9 Hz, 4H), 5.72 (s, 2H). ^{13}C NMR (100 MHz, DMSO-d_6): 166.94, 151.05, 141.21, 139.38, 138.89, 132.73, 131.99, 131.78, 130.75, 128.39, 122.48.

Synthesis of MFM-172

4',4'''-(4-Methylpyridine-3,5-diyl)bis(([1,1'-biphenyl]-3,5-dicarboxylic acid)) (H_4L^2) (12 mg, 0.017 mmol) and $Cu(NO_3)_3·2.5H_2O$ (24 mg, 0.081 mmol) were added to a mixture of DMF (2.5 mL) and 3 drops of *conc*. HNO_3. The solution was heated in 8 mL Wheaton vials at 353K for 1 day. The blue crystals were separated by filtration, washed with hot DMF, acetone exchanged and dried in air (9.5 mg, 80%). $[Cu_2(C_{34}H_{19}NO_8)(H_2O)]$, IR (ATR) cm^{-1}: 3064 (C-H), 1640 (COO, ν_{as}), 1424 (COO, ν_s) and 831 (CH_3); Elemental analysis for $[Cu_2(C_{34}H_{19}NO_8)]$ (% calculated/found): C 58.6/58.4 H 2.7/2.6 N 2.0/1.8 Cu18.2/18.4.

Synthesis of MFM-174

4',4'''-(4-Aminopyridine-3,5-diyl)bis(([1,1'-biphenyl]-3,5-dicarboxylic acid)) (H_4L^3) (12 mg, 0.017 mmol) and $Cu(NO_3)_3·2.5H_2O$ (24 mg, 0.081 mmol) were added to a mixture of DMF (2.5 mL) and 3 drops of *conc*. HNO_3. The solution was heated in 8 mL Wheaton vials at 353K for 1 day. The blue crystals were separated by filtration, washed with hot DMF, acetone exchanged and dried in air (9.5 mg, 80%). $[Cu_2(C_{33}H_{18}N_2O_8)(H_2O)]$, IR (ATR) cm^{-1}: 3514, 3414 (N-H), 3032 (C-H), 1640 (COO, ν_{as}) and 1424 (COO, ν_s); Elemental analysis for $[Cu_2(C_{33}H_{18}N_2O_8)]$ (% calculated/found): C 56.8/57.0 H 2.6/2.8 N 4.0/3.9 Cu18.2/18.0.

1.2 BET measurements

The acetone-exchanged MOF samples were dried in air and treated at 150 °C and 10^{-10} bar for 12 h to yield the fully desolvated samples, which were then loaded in 3-flex instruments (Micrometrics company) for porosity characterization. The BET surface areas and total pore volume were calculated using the N_2 isotherms measured at 77 K.

1.3 Powder X-ray diffraction

Powder X-Ray diffraction (PXRD) data were collected in flat plate mode over the 2θ range 5-30° on an X'pert multipurpose Diffractometer using Cu-Kα radiation (λ = 1.54056 Å) at 45 kV and 40 mA.

1.4 SEM-EDX experiments

SEM measurements were undertaken on a Quanta 650 at a working voltage of 20 kV with a scale bar up to 10 microns. Cu and I element were detected by energy-dispersive X-ray spectroscopy.

1.5 Iodine adsorption

Prior to adsorption, the acetone-exchanged samples were heated under vacuum (1 mbar) for 8 h at 150 °C. The activated MOF sample (100 mg) was transferred into a vessel containing solid I_2 (3.0 g) in an open vial. The vessel was charged with dry N_2 at atmospheric pressure to allow adsorption of I_2 vapour by the MOF under the same I_2 pressure for all samples. To determine the background, an empty glass vial was loaded into the vessel. The time-resolved I_2 adsorption profiles of MFM-170, MFM-174, NJU-Bai20 and for the blank vial are shown in Figure S15. The vessel was sealed and heated at 80 °C for 0.5-58 h under N_2 to allow adsorption of I_2 into the desolvated MOFs. The I_2-loaded samples were cooled to room temperature and collected for further analysis. For the cycling experiment, the sample was reactivated under vacuum and 150 °C between each cycle. Physisorbed I_2 can be fully removed by reactivation by heating at 80-180 °C; chemisorbed I_2 bound to the framework cannot be removed in this way and requires higher temperatures.

1.6 Thermogravimetric analysis

Thermogravimetric analysis (SDT650 TA Instruments company) was used to determine the uptake of adsorbed

I_2 molecules within these MOF materials. Samples were heated from room temperature to 600 °C at a rate of 5 °C min^{-1} under a flow of air. The TGA plots of activated MOF samples were obtained by *in situ* activation on TA instrument. The bare sample (~10 mg) was loaded onto the pan and the sample was heated to 150 °C under N_2 for 2 h, and the temperature was decreased to room temperature. After *in situ* activation, the temperature was increased to 600 °C at a rate of 5 °C min^{-1} under a flow of air.

1.7 X-ray photoelectron spectroscopy (XPS)

XPS spectra were measured using a Kratos Axis Ultra instrument equipped with a monochromatic Al Kα X-ray source (E = 1486.6 eV). A charge neutraliser was used to minimise charging and spectra were aligned to the binding energy scale relative to the hydrocarbon C-C/C-H peak at 284.8 eV. Spectra were fitted using the CASA XPS software using Voigt-like peak shapes. Spin-orbit splitting ratios and splitting energies were constrained to obtain physically meaningful fits.

1.8 MAS NMR spectroscopy

Magic angle spinning (MAS) NMR spectra were recorded using a Bruker 9.4 T (400 MHz ^{1}H Larmor frequency) AVANCE III spectrometer equipped with a 4 mm HFX MAS probe. Samples were treated and packed into 4 mm o.d. zirconia rotors under inert conditions and sealed with a Kel-F rotor cap. Experiments were acquired at ambient temperature using a MAS frequency of 12 kHz. ^{1}H-pulses of 100 kHz were used for the ^{1}H MAS NMR spectra that employed a Hahn-echo sequence with an inter-pulse delay of one rotor period, giving a total echo time of 0.167 ms. 64 transients were co-added for each spectrum, with a recycle delay of 0.1 s used between each scan. {^{1}H-}^{13}C cross-polarisation (CP)MAS NMR spectra were acquired with ^{1}H-pulses of 100 kHz and ^{13}C spin-locking at ~50 kHz that was applied for 1 ms, with corresponding ramped (70-100 %) ^{1}H spin-locking at ~75 kHz with 100 kHz of SPINAL-64[4] heteronuclear ^{1}H decoupling used throughout. A Hahn-echo sequence with an inter-pulse delay of one rotor period was used after CP in the CPMAS NMR experiments where between 1024 and 4800 transients were co-added for each spectrum, with a recycle delay of 1 s used between each scan.

1.9 Synchrotron single crystal X-ray diffraction

Data collection. X-ray data for I_2@MFM-170, I_2@MFM-172, I_2@MFM-174 and I_2@NJU-Bai20 were collected at 100-150 K using synchrotron radiation at the single crystal X-ray diffraction beamline I19 at Diamond Light Source[5], equipped with a Pilatus 2M detector and an Oxford Cryosystems nitrogen flow gas system. Data were measured using GDA suite of programs.

Crystal structure determinations and refinements. X-ray data were processed and reduced using CrysAlisPro and dials[6, 7]. Absorption corrections were performed using empirical methods (SCALE3 ABSPACK and SADABS) based upon symmetry-equivalent reflections combined with measurements at different azimuthal angles. The crystal structure was solved and refined against all F^2 values using the SHELX and Olex2 suite of programmes[8-10]. Hydrogen atoms were placed in calculated positions and refined using idealised geometries and assigned fixed isotropic displacement parameters.

Crystal structure of I_2@MFM-170: All atoms were refined anisotropically with the exception of the highly disordered I_2 molecules. The structure contained disordered phenyl moieties which were constrained to have an ideal geometry, and were modelled over two sites with 50% occupancies related through an inversion center.

I-I bond distances were restrained using SHELX distance fix (DFIX) command. The occupancies of the coordinated water molecule and the I_2 molecules were refined, and atomic displacement parameters were restrained using similar and rigid body approach using SHELX SIMU and RIGU commands. The swap parameter was refined to stabilise the molecules modelled in the pores. Alternatively, solvent mask protocol was applied to the crystals structure without any I_2 in the pores to obtain an electron count consistent with 3 I_2 molecules per formula unit. The difference electron density map obtained after applying the solvent mask protocol showed a large concentration of electron density in the pore close to the phenyl groups. The mismatch between the amounts of iodine obtained in the model and from the solvent mask protocol arise from the proximity of the disordered phenyl moieties with the electron density corresponding to a disordered I_2. As consequence, the solvent mask protocol could not take into account all the remaining electron density corresponding to I_2.

Crystal structure of I_2@MFM-172: Data resolution was found to be 1.1 Å. All atoms were refined anisotropically with the exception of the disordered I_2 molecules and phenyl moieties. I-I bond distances were restrained using SHELX distance fix (DFIX) command, and the U_{iso} parameters were constrained to be 0.3. The occupancies of the coordinated water molecule and the I_2 molecules were refined. Alternatively, solvent mask protocol was applied to the crystal structure without any I_2 in the model to obtain 2 I_2 molecules per formula unit.

Crystal structure of I_2@MFM-174: Data resolution was found to be 1.15 Å. All atoms were refined anisotropically with the exception of the disordered I_2 molecules and 4-aminopyridyl moieties. I-I bond distances were restrained using SHELX distance fix (DFIX) command, and U_{iso} parameters were constrained to be 0.3. The occupancies of the coordinated water molecule and the I_2 molecules were refined. The ill-shaped remaining electron density of the pores was accounted using solvent mask protocol implemented in Olex2. The number of electrons found was 612 per unit cell corresponding to 0.25 I_2 molecules per formula unit. Alternatively, solvent mask protocol was applied to the crystals structure without any I_2 in the model to obtain 2.24 I_2 molecules per formula unit.

Crystal structure of I_2@NJU-Bai20: All atoms were refined anisotropically with the exception of the disordered I_2 molecules and water molecules. I-I bond distances were restrained using SHELX distance fix (DFIX) command. U_{iso} parameters were constrained to be 0.3, and the occupancies of the coordinated water and I_2 molecules were refined. Alternatively, solvent mask protocol was applied to the crystals structure without any I_2 in the model to obtain 3.36 I_2 molecules per formula unit.

1.10 γ-Irradiation

The FTS Model 812 γ-irradiator is designed specifically to support a wide range of research applications in order to understand the mechanistic effects of γ radiation on exposed materials. Here, the effects of γ radiation on waste storage media including MFM-170, MFM-174 and NJU-Bai20 were investigated. The ^{60}Co sources are arranged in a circle allowing for a uniform dose to the materials being irradiated. The samples were loaded in the sample irradiation chamber (200 x 250 x 270 mm), and a chamber dose rate of 340 Gy/hr was applied for 215 hours.

1.11 Raman spectroscopy

Raman spectra were obtained using a Renishaw inVia microscope with a 532 nm with an acquisition time of 90 s and accumulated for 3 cycles.

2. Data and views of crystal structures

Table S1. Data for crystal structures of MFM-172 and MFM-174

Identification code	**MFM-172**	**MFM-174**
Empirical formula	$C_{34}H_{26}Cu_2NO_{8.78}$	$C_{33}H_{18}Cu_2N_2O_9$
Formula weight	716.16	711.56
Temperature/K	293	293
Crystal system	cubic	cubic
Space group	Im-3m	Im-3m
a/Å	33.5367(3)	33.4232(6)
b/Å	33.5367(3)	33.4232(6)
c/Å	33.5367(3)	33.4232(6)
α/°	90	90
β/°	90	90
γ/°	90	90
Volume/$Å^3$	37719.2(9)	37337.4(2)
Z	24	24
ρ_{calc} g/cm^3	0.757	0.759
μ/mm^{-1}	0.249	1.093
F(000)	8766.0	8592.0
Crystal size/mm^3	0.1 × 0.1 × 0.1	0.0 × 0.1 × 0.1
Radiation	Synchrotron (λ = 0.4859)	CuKα (λ = 1.54184)
2Θ range for data collection/°	3.106 to 35.366	3.738 to 153.65
Index ranges	$-41 \le h \le 41$, $-41 \le k \le 38$, $-41 \le l \le 41$	$-40 \le h \le 25$, $-37 \le k \le 30$, $-39 \le l \le 40$
Reflections collected	247666	68163
Independent reflections	3630 [R_{int} = 0.1484, R_{sigma} = 0.0273]	3647 [R_{int} = 0.0482, R_{sigma} = 0.0175]
Data/restraints/parameters	3630/139/143	3647/18/143
Goodness-of-fit on F^2	1.042	1.068
Final R indexes [I>=2σ (I)]	R_1 = 0.0342, wR_2 = 0.0939	R_1 = 0.0635, wR_2 = 0.1908
Final R indexes [all data]	R_1 = 0.0366, wR_2 = 0.0953	R_1 = 0.0731, wR_2 = 0.2021
Largest diff. peak/hole / e $Å^{-3}$	0.28/-0.52	0.62/-0.36

Table S2. Comparison of unit cells and BET surface area

MOFs	Crystal system	a (Å)	α	V ($Å^3$)	BET (m^2/g)	Fractional porosity	Pore volume (cm^3/g)
NJU-Bai21	***I*** m-3 m	30.41	90	28,130	1372	56%	0.63
NJU-Bai20	***I*** m-3 m	31.11	90	30,109	2081	67%	0.83
MFM-170	***I*** m-3 m	33.47	90	37,505	2408	61%	0.82
MFM-172	***I*** m-3 m	33.41	90	37,505	2076	60%	0.79
MFM-174	***I*** m-3 m	33.41	90	37,505	2251	62%	0.81

Table S3. Data for crystal structures of I_2-loaded MOFs

Identification code	**I_2@MFM-170**	**I_2@MFM-172**	**I_2@MFM-174**	**I_2@NJU-Bai20**
Empirical formula	$[4.39I_2 \cdot Cu_2(C_{33}H_{17}NO_8) \cdot (H_2O)_{0.79})]$	$[1.6I_2 \cdot Cu_2(C_{34}H_{19}NO_8) \cdot (H_2O)]$	$[1.84I_2 \cdot Cu_2(C_{33}H_{18}N_2O_8) \cdot (H_2O)]$	$[1.82I_2 \cdot Cu_2(C_{25}H_9N_2O_8) \cdot (H_2O)_{0.96}]$
Formula weight	1810.41	1132.26	1180.68	1051.96
Temperature/K	150.00	100	100	100.0
Crystal system	cubic	cubic	cubic	cubic
Space group	Im-3m	Im-3m	Im-3m	Im-3m
a/Å	33.43010(10)	33.4070(6)	33.2328(4)	30.9926(3)
b/Å	33.43010(10)	33.4070(6)	33.2328(4)	30.9926(3)
c/Å	33.43010(10)	33.4070(6)	33.2328(4)	30.9926(3)
α/°	90	90	90	90
β/°	90	90	90	90
γ/°	90	90	90	90
Volume/Å^3	37360.5(3)	37283(2)	36702.9(13)	29769.7(8)
Z	24	24	24	24
ρ_{calc} g/cm^3	2.080	1.210	1.282	1.408
μ/mm^{-1}	5.196	2.174	2.391	2.917
F(000)	21008.0	12874.0	13322.0	11633.0
Crystal size/mm^3	0.2 × 0.2 × 0.2	0.1 × 0.1 × 0.1	0.04 × 0.02 × 0.02	0.1 × 0.1 × 0.1
Radiation	Synchrotron (λ = 0.6889)	Synchrotron (λ = 0.6889)	Synchrotron (λ = 0.6889)	Synchrotron (λ = 0.6889)
2Θ range for data collection/°	1.67 to 50.184	2.894 to 36.49	2.91 to 34.834	3.12 to 49.032
Index ranges	$-38 \leqslant h \leqslant 36, -37 \leqslant k \leqslant 40, -37 \leqslant l \leqslant 38$	$-12 \le h \le 30, -30 \le k \le 27, -30 \le l \le 28$	$-28 \le h \le 28, -28 \le k \le 28, -28 \le l \le 28$	$-37 \le h \le 35, -33 \le k \le 37, -35 \le l \le 33$
Reflections collected	79698	24835	59734	105253
Independent reflections	3256 [R_{int} = 0.0495, R_{sigma} = 0.0222]	1455 [R_{int} = 0.1344, R_{sigma} = 0.0404]	1258 [R_{int} = 0.1575, R_{sigma} = 0.0327]	2608 [R_{int} = 0.1356, R_{sigma} = 0.0312]
Data/restraints/parameters	3256/177/241	1455/7/151	1258/62/140	2608/10/144
Goodness-of-fit on F^2	2.092	2.126	2.721	1.583
Final R indexes [I>=2σ (I)]	R_1 = 0.1753, wR_2 = 0.4554	R_1 = 0.1718, wR_2 = 0.4621	R_1 = 0.1988, wR_2 = 0.4886	R_1 = 0.1478, wR_2 = 0.3762
Final R indexes [all data]	R_1 = 0.1961, wR_2 = 0.4731	R_1 = 0.1971, wR_2 = 0.4854	R_1 = 0.2079, wR_2 = 0.5142	R_1 = 0.1690, wR_2 = 0.3902
Largest diff. peak/hole / e Å^{-3}	2.19/-1.97	1.83/-1.54	1.19/-0.80	1.95/-1.40

Table S4. Summary of binding sites in MFM-17x and NJU-Bai20 (occupancy: I_2/{Cu_2})

Cage	Binding site	MFM-170	MFM-172	MFM-174	NJU-Bai20
**Cage C occu**	C-I	0.341	0.233	0.126	0.175
	C-II	0.468	0.215	0.083	0.135
	C-III	0.292	0.096	-	-
	Total occupancy in cage C	1.101	0.545	0.209	0.311
**Cage B occu**	B-I	0.783	0.321	0.518	0.239
	B-II	0.578	0.306	0.325	0.217
	B-III	0.414	0.278	0.303	0.184
	B-IV	0.409	0.151	0.231	0.174
	B-V	0.378	-	0.136	0.137
	B-VI	0.365	-	0.117	-
	Total occupancy in cage B	2.927	1.055	1.630	0.950
**Cage A occu**	A-I	0.366	-	-	0.307
	A-II	-	-	-	0.149
	A-III	-	-	-	0.104
	Total occupancy in cage A	0.366	-	-	0.559
**Disorder**		0.55[a]	0.40	0.40	1.54
	**Total occu**	**4.94**	**2.00**	**2.24**	**3.36**

a. The two iodine atoms at B^{V}, B^{VI} and A^{I} show different occupancies. The atom with the lower occupancy was included at binding site and the residue was included in disorder.

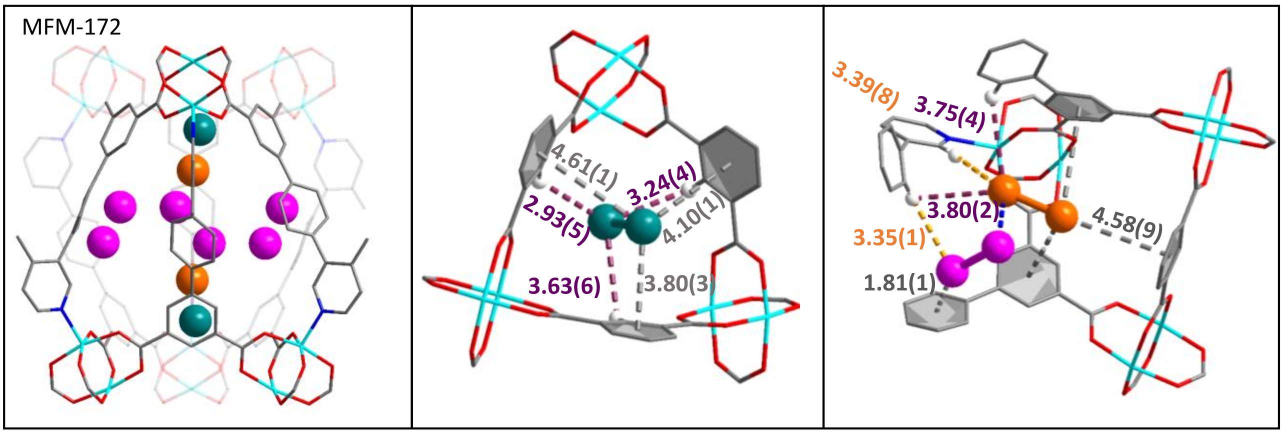

Figure S1. Views of structures of cage C in I_2-loaded MFM-172. Left: view of I_2 binding sites in cage C of MFM-172 (Site C-I: Teal; Site C-II: pink; Site C-III: orange); middle: view of intermolecular interactions between $I_2^{C\text{-}I}$ and MFM-172; right: view of intermolecular interactions between $I_2^{C\text{-}II}$, $I_2^{C\text{-}III}$ and MFM-172 (C, grey; O, red; Cu, cyan; N, blue; H, white) all unit is Å.

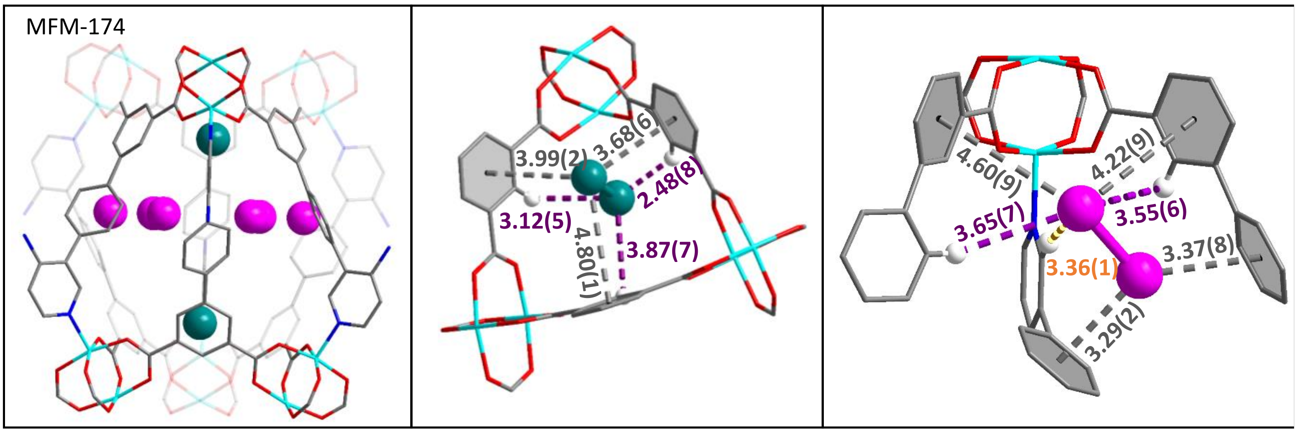

Figure S2. Views of structures of cage C in I_2-loaded MFM-174. Left: view of I_2 binding sites in cage C of MFM-174 (Site C-I: Teal; Site C-II: pink); middle: view of intermolecular interactions between $I_2^{C\text{-}I}$ and MFM-174; right: view of intermolecular interactions between $I_2^{C\text{-}II}$ and MFM-174 (C, grey; O, red; Cu, cyan; N, blue; H, white) all unit is Å.

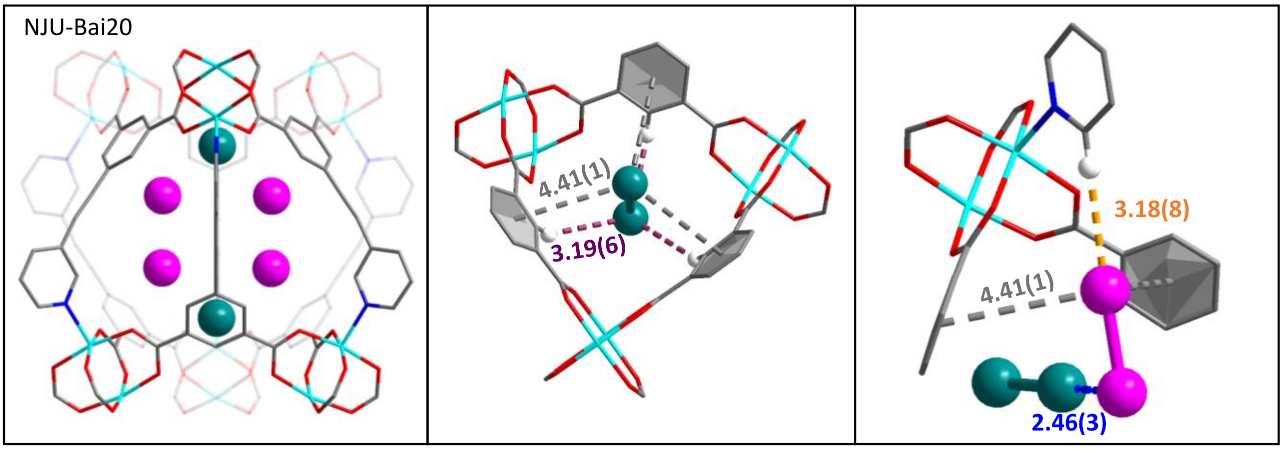

Figure S3. Views of structure of cage C in I_2-loaded NJU-Bai20. Left: view of I_2 binding sites in cage C of NJU-Bai20 (Site C-I: Teal; Site C-II: pink); middle: view of intermolecular interactions between $I_2^{C\text{-}I}$ and NJU-Bai20; right: view of intermolecular interactions between $I_2^{C\text{-}II}$ and NJU-Bai20 (C, grey; O, red; Cu, cyan; N, blue; H, white; all unit is in Å).

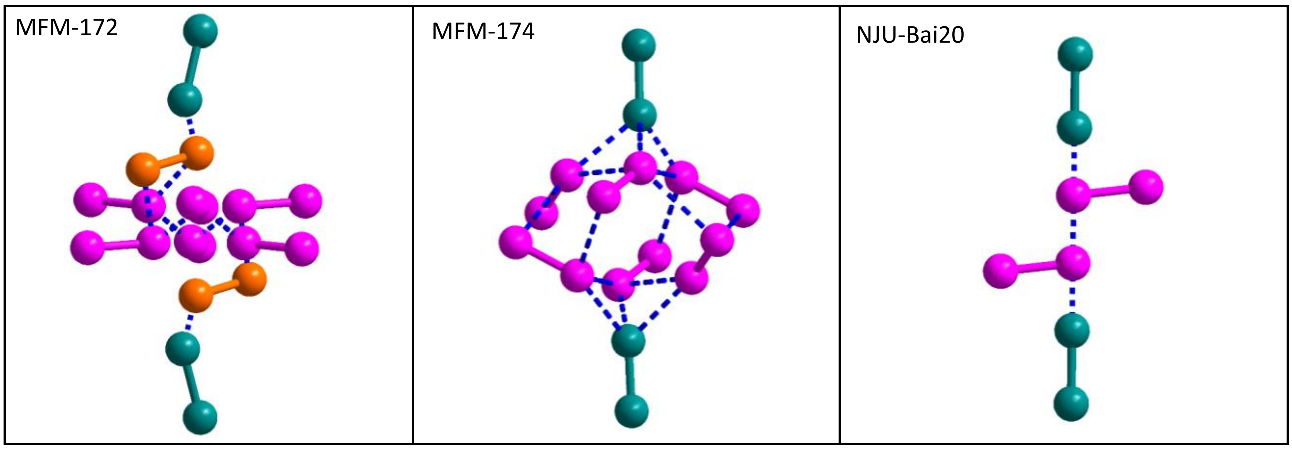

Figure S4. Views of I_2 packing in cage C of MFM-172 (left), MFM-174 (middle) and NJU-Bai20 (right).

MFM-170

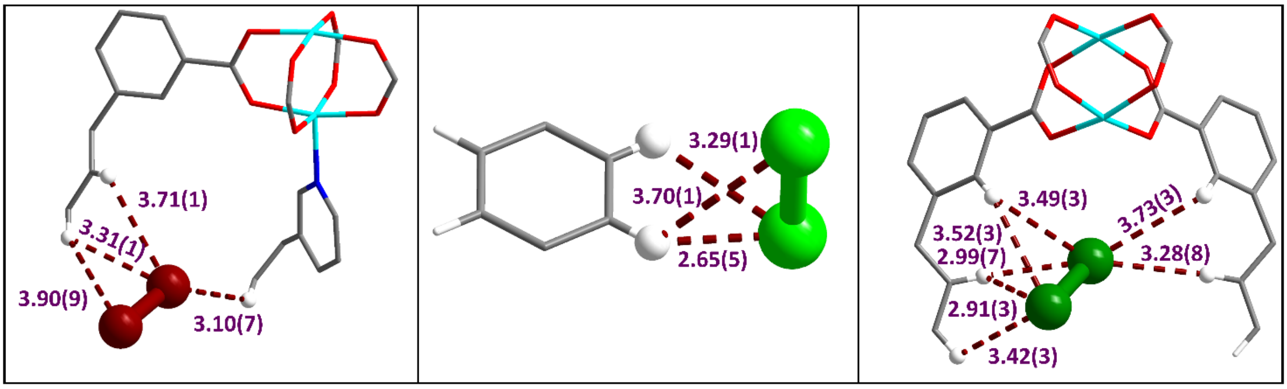

Figure S5. Views of structure of cage B in MFM-170. Left: view of intermolecular interactions between $I_2^{B\text{-}III}$ and MFM-170; middle: view of intermolecular interactions between $I_2^{B\text{-}V}$ and MFM-170; right: view of intermolecular interactions between $I_2^{B\text{-}VI}$ and MFM-170 (C, grey; O, red; Cu, cyan; N, blue; H, white; all unit is in Å).

MFM-172

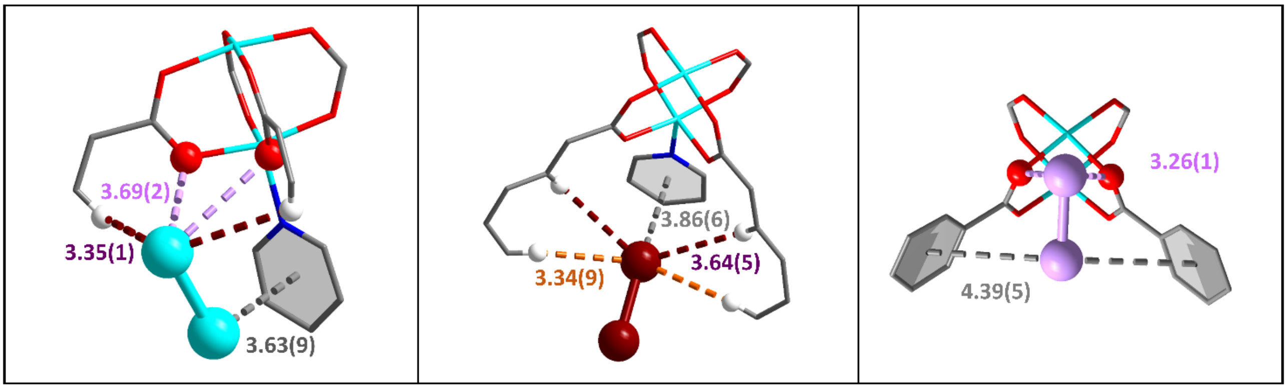

Figure S6. Views of structures of cage B in MFM-172. Left: view of intermolecular interactions between $I_2^{B\text{-}II}$ and MFM-172; middle: view of intermolecular interactions between $I_2^{B\text{-}III}$ and MFM-172; right: view of intermolecular interactions between $I_2^{B\text{-}IV}$ and MFM-172 (C, grey; O, red; Cu, cyan; N, blue; H, white; all unit is in Å).

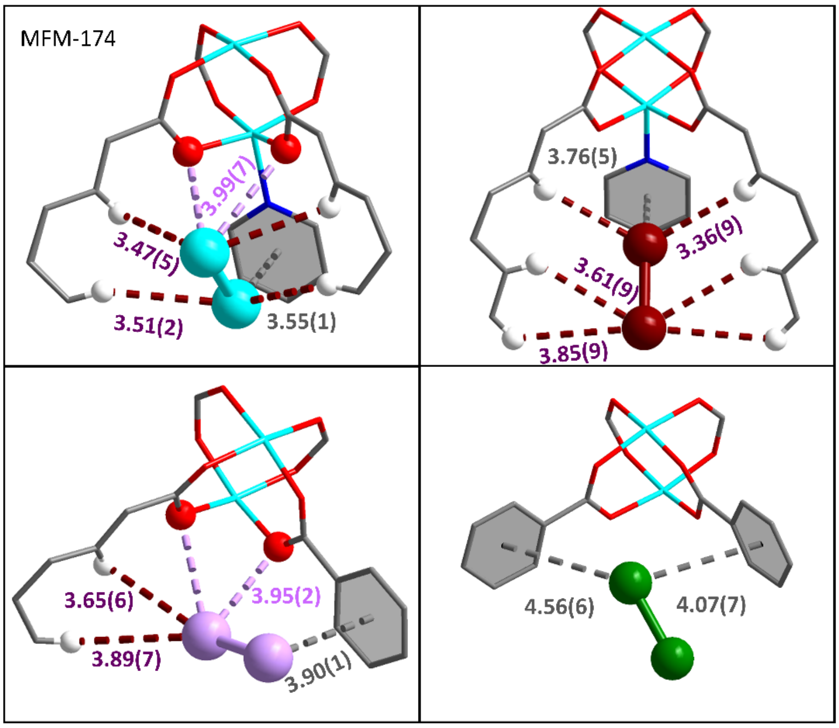

Figure S7. Views of structure of cage B in MFM-174. Top left: view of intermolecular interactions between $I_2^{B\text{-}II}$ and MFM-174; top right: view of intermolecular interactions between $I_2^{B\text{-}III}$ and MFM-174; bottom left: view of intermolecular interactions between $I_2^{B\text{-}IV}$ and MFM-174; bottom right: view of intermolecular interactions between $I_2^{B\text{-}VI}$ and MFM-174 (C, grey; O, red; Cu, cyan; N, blue; H, white; all unit is in Å.

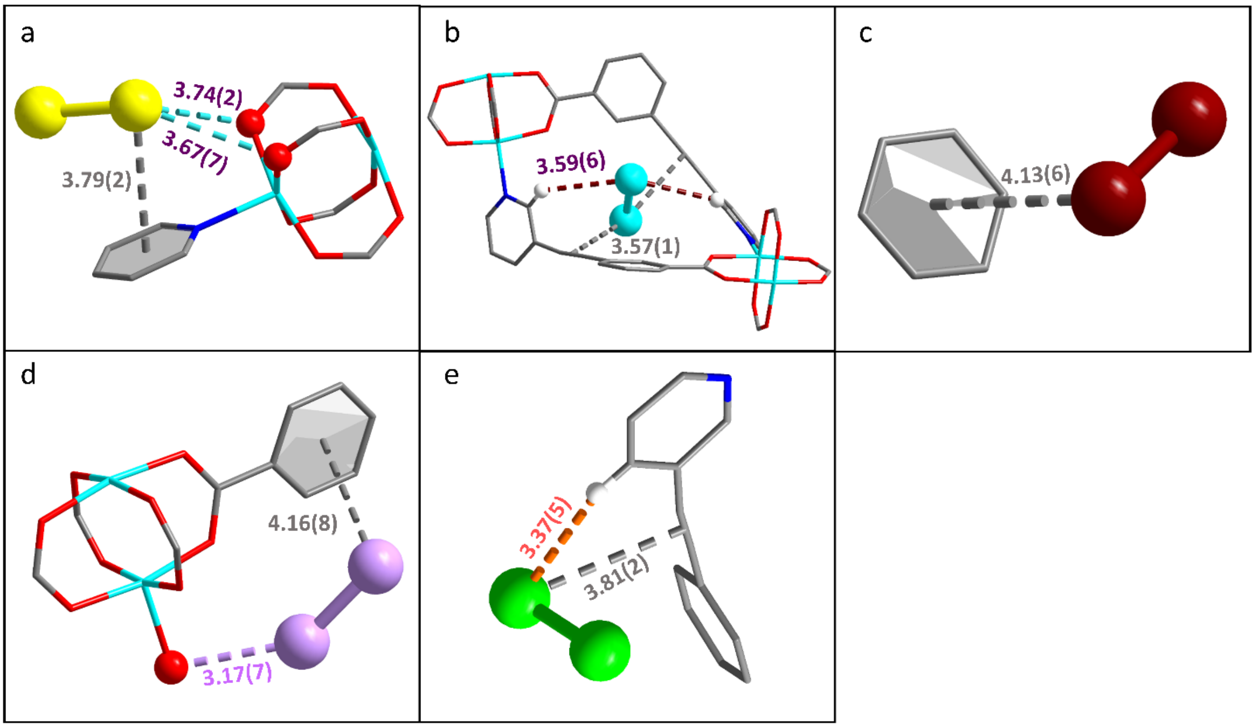

Figure S8. Views of structure of cage B in NJU-Bai20. (a) View of intermolecular interactions between $I_2^{B\text{-}I}$ and NJU-Bai20; (b) view of intermolecular interactions between $I_2^{B\text{-}II}$ and NJU-Bai20; (c) view of intermolecular interactions between $I_2^{B\text{-}III}$ and NJU-Bai20; (d) view of intermolecular interactions between $I_2^{B\text{-}IV}$ and NJU-Bai20; (e) view of intermolecular interactions between $I_2^{B\text{-}V}$ and NJU-Bai20 (C, grey; O, red; Cu, cyan; N, blue; H, white; all unit is in Å).

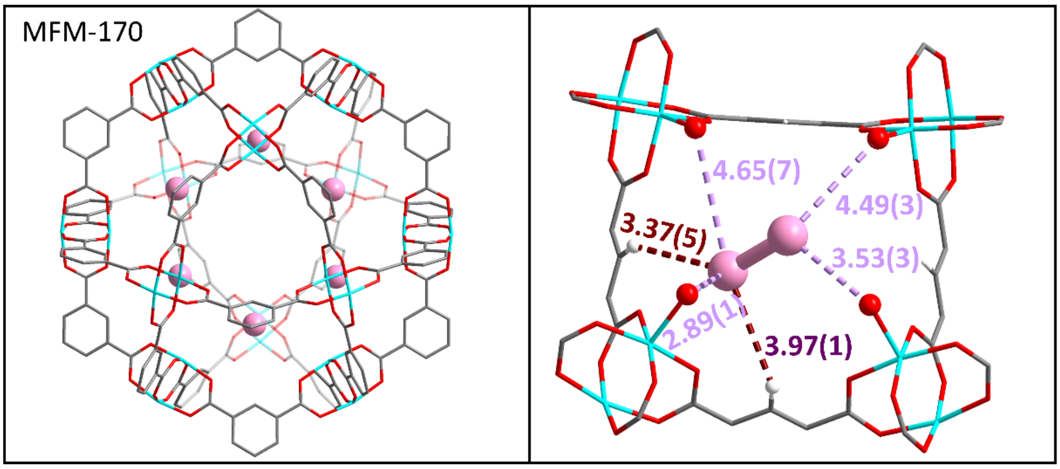

Figure S9. Views of structure of cage A in MFM-170. Left: View of I_2 binding sites in cage A of MFM-170; right: view of intermolecular interactions between $I_2^{A\text{-}I}$ and MFM-170 (C, grey; O, red; Cu, cyan; N, blue; H, white; all unit is in Å).

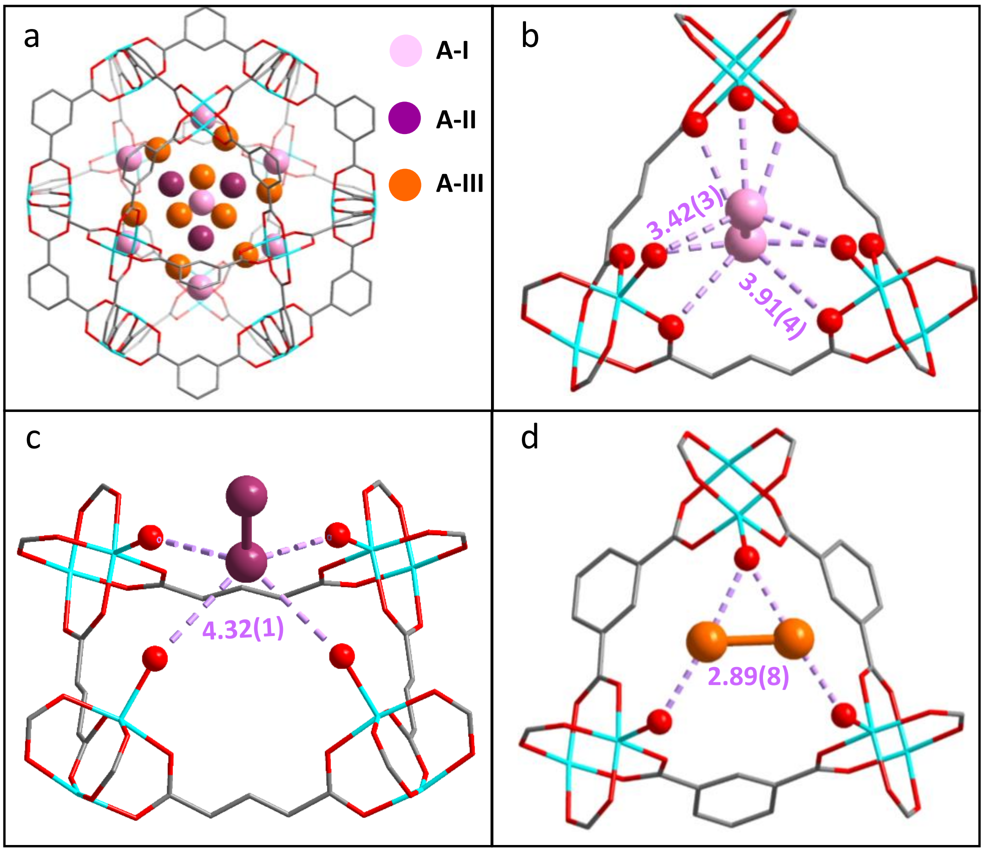

Figure S10. Views of crystal structures of I_2-loaded NJU-Bai20. Views of (a) I_2 binding sites in cage A of NJU-Bai20; intermolecular interactions between (b) $I_2^{A\text{-}I}$ and NJU-Bai20, (c) $I_2^{A\text{-}II}$ and NJU-Bai20, and (d) $I_2^{A\text{-}III}$ and NJU-Bai20 (C, grey; O, red; Cu, cyan; N, blue; H, white; all units are in Å).

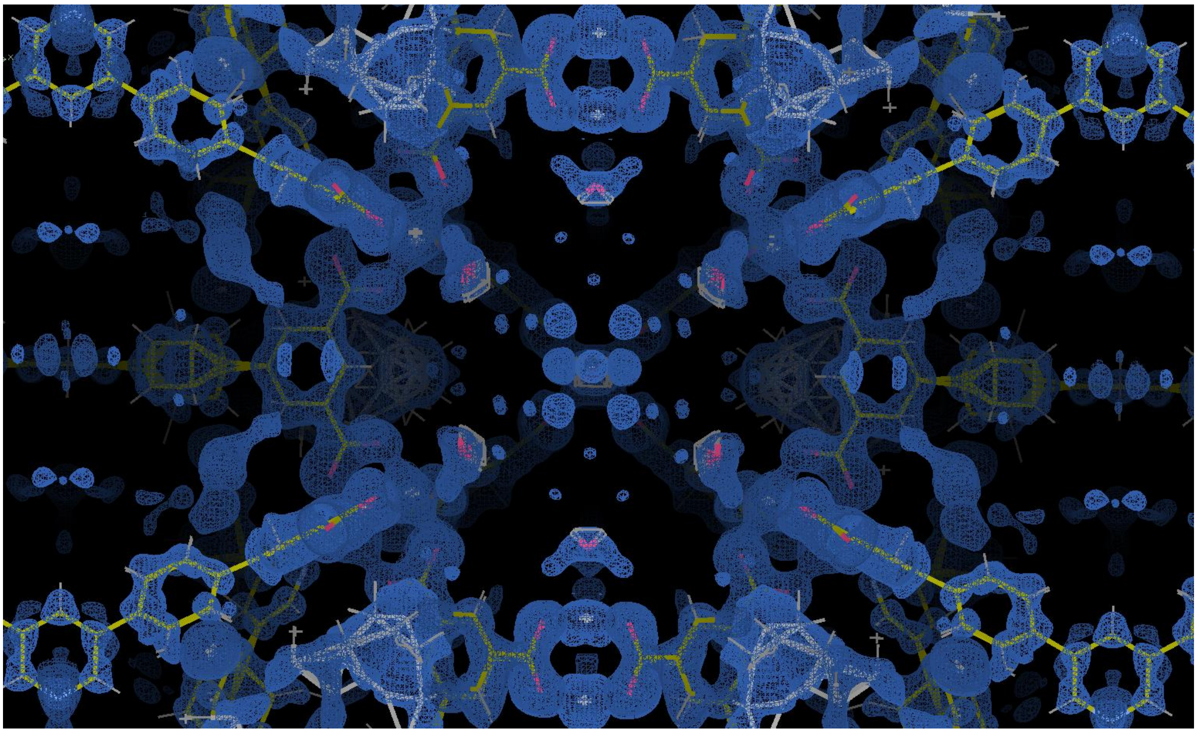

Figure S11. 2Fo-Fc electron density map representation of I_2-loaded MFM-170. Electron density map pictures (4 msd) are represented in blue, carbon atoms are represented in green, oxygen in light red, iodine in grey and copper as white crosses. 2Fo-Fc electron density maps were obtained using Phenix software. Initially, the reflections file (.mtz) obtained from CrysAlisPro was edited to include the map coefficients. The maps were calculated from the edited reflections file and the model (pdb file) obtained from structure solution software (OLEX 2) using phenix.maps software.

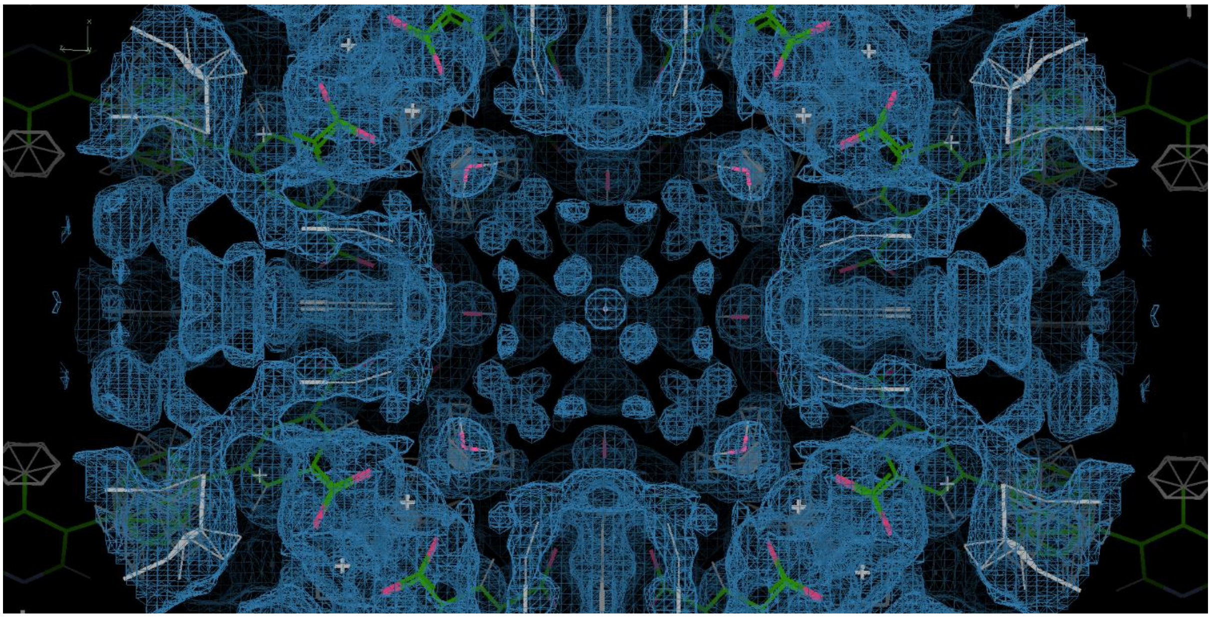

Figure S12. 2Fo-Fc electron density map representation of I_2-loaded MFM-172. Electron density map pictures (4 msd) are represented in blue, carbon atoms are represented in green, oxygen in light red, iodine in grey and copper as white crosses. 2Fo-Fc electron density maps were obtained using Phenix software. Initially, the reflections file (.mtz) obtained from CrysAlisPro was edited to include the map coefficients. The maps were calculated from the edited reflections file and the model (pdb file) obtained from structure solution software (OLEX 2) using phenix.maps software.

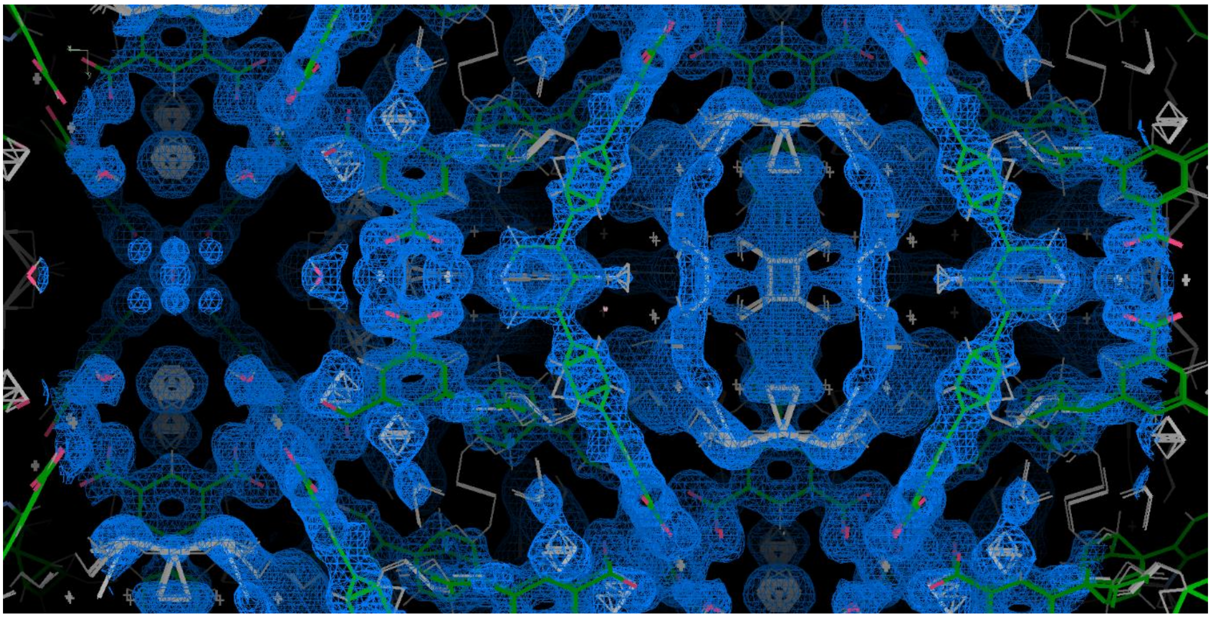

Figure S13. 2Fo-Fc electron density map representation of I_2-loaded MFM-174. Electron density map pictures (4 msd) is represented in blue, carbon atoms and nitrogen are represented in green, oxygen in light red, iodine in grey and copper as white crosses. 2Fo-Fc electron density maps were obtained using Phenix software. Initially, the reflections file (.mtz) obtained from CrysAlisPro was edited to include the map coefficients. The maps were calculated from the edited reflections file and the model (pdb file) obtained from structure solution software (OLEX 2) using phenix.maps software.

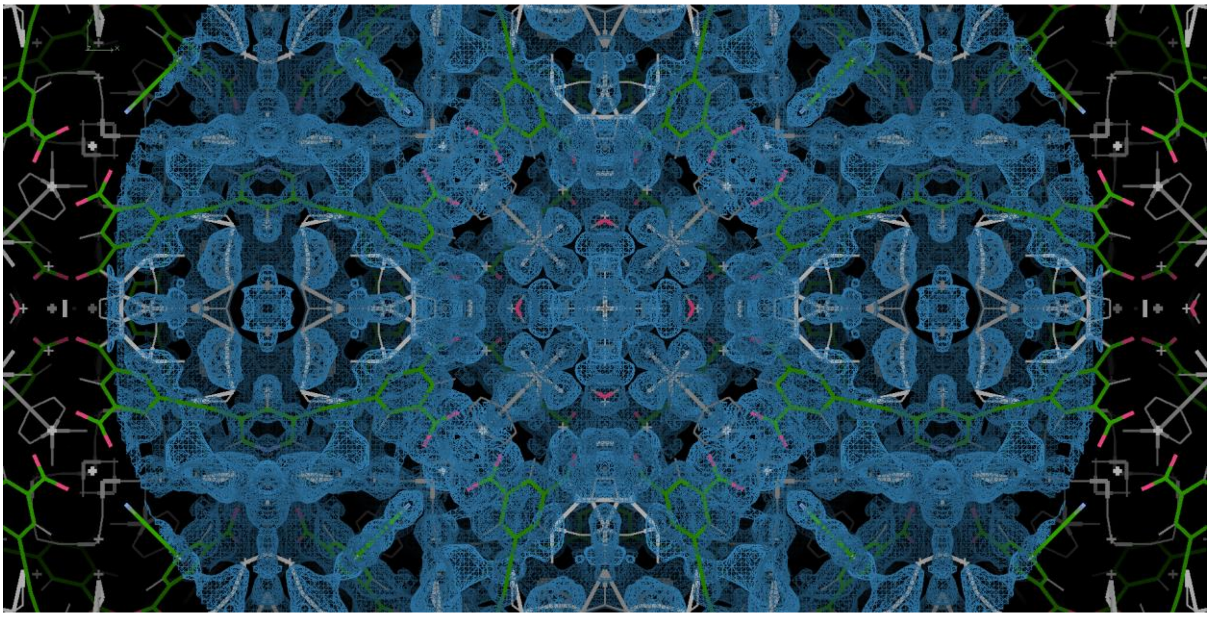

Figure S14. 2Fo-Fc electron density map representation of I_2-loaded NJU-Bai20. Electron density map pictures is represented in blue, carbon atoms and nitrogen are represented in green, oxygen in light red, iodine in grey and copper as white crosses. 2Fo-Fc electron density maps were obtained using Phenix software. Initially, the reflections file (.mtz) obtained from CrysAlisPro was edited to include the map coefficients. The maps were calculated from the edited reflections file and the model (pdb file) obtained from structure solution software (OLEX 2) using phenix.maps software.

3. Analysis of I_2-adsorption

Table S5. Element analysis and ICP-AES results for I_2@MFM-170, I_2@MFM-174 and I_2@NJU-Bai 20 after heating at 300 °C for 2 h to remove physisorbed I_2.

	C	H	N	Cu	I	Residue I_2
I_2@MFM-170	34.96	2.09	1.43	12.07	trace	trace
I_2@MFM-174	35.94	1.8	1.93	12.26	38.64	0.56 g/g
I_2@NJU-Bai20	31.56	1.52	1.39	13.34	17.69	0.28 g/g

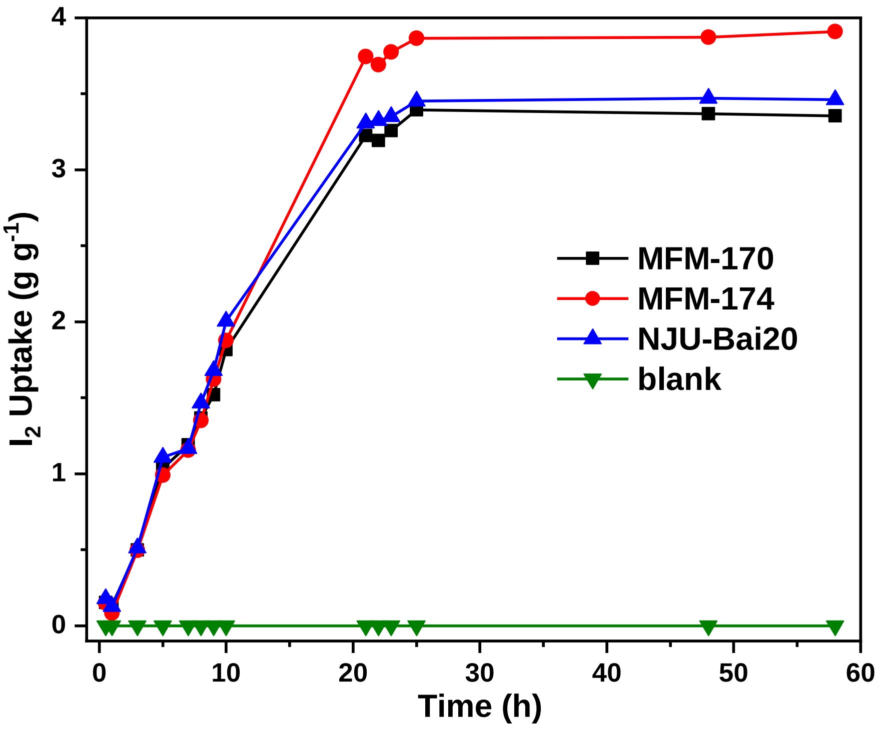

Figure S15. The time-resolved I_2 adsorption profiles of MFM-170, MFM-174, NJU-Bai-20 and a blank vial (as the background).

Table S6. Summary of iodine adsorption in MOFs *via* vapor diffusion

MOFs	pore volume ($cm^3\ g^{-1}$)	Uptake ($g\ g^{-1}$)	packing density ($g\ cm^{-3}$)	ref
NJU-Bai21	0.63	1.79	2.84	this work
NJU-Bai20	0.83	3.47	4.18	this work
MFM-170	0.82	3.37	4.11	this work
MFM-172	0.79	2.91	3.68	this work
MFM-174	0.81	3.91	4.83	this work
Cu-BTC	0.74	1.75	2.36	11
[Cu_4I_4-MOF]	0.31	0.14	0.45	12
[$Fe_3(HCOO)_6$]	0.15	0.49	3.27	13
Ca(sdb)	0.62	0.26	0.42	14
Ca(tcpb)	0.84	0.43	0.51	14
ZIF-8	0.66	1.25	1.89	15
[Zn_3(DL-lac)$_2$(pybz)$_2$]	0.41	1.01	2.46	16
[Zn_2(μ_4-ao$_2$btc)(μ-pbix)$_2$]	0.07	0.2	2.86	17
[$Zr_6O_4(OH)_4$ (sdc)$_6$]	1.33	1.07	0.8	18
[$Zr_6O_4(OH)_4$ (edb)$_6$]	1.39	1.8	1.29	18
[$Zr_6O_4(OH)_4$ (bdb)$_6$]	1.7	1.8	1.06	18
[$Zr_6O_4(OH)_4$ (peb)$_6$]	1.16	2.79	2.41	18
MFM-300(Sc)	0.5	1.54	3.08	19
MFM-300(In)	0.41	1.16	2.83	19
MFM-300(Fe)	0.46	1.29	2.8	19
MFM-300(Al)	0.37	0.94	2.54	19
UPC-158	0.93	1.78	1.91	20
UPC-158-HF	0.96	2.19	2.28	20
UPC-158-HCl	0.99	2.92	2.95	20
UPC-158-HBr	0.93	2.75	2.96	20
UPC-158-HCl	0.85	2.59	3.05	20
MBM	0.74	0.98	1.32	21
HKUST-1@PES	0.31	0.38	1.21	22
MIL-53-SH(Al)	0.07	0.33	4.7	23
Zn_2(tptc)(apy)	0.6	2.16	3.6	24
MOF-808	0.82	2.18	2.65	25
NU-1000	1.27	1.45	1.14	25
MOF-867	1.12	0.88	0.78	25
UiO-66	0.53	0.66	1.24	25
UiO-67	1.17	0.53	0.45	25
PCN-333(Al)	2.97	4.42	1.49	26
UiO-66-FA	0.73	2.25	3.08	27

Table S7. Summary of iodine adsorption in polymers, COFs and activated carbon *via* vapor diffusion

Materials	pore volume (cm^3 g^{-1})	Uptake (g g^{-1})	packing density (g cm^{-3})	ref
**COFs**				
QTD-COF-V	-	6.29	-	28
TPB-DMTP	1.28	6.20	4.84	29
TTA-TTB	1.01	4.95	4.90	29
COF-DL229	0.64	4.7	7.34	30
TPT-DHBD	0.30	5.43	18.1	31
SIOC-COF-7	0.41	4.81	11.7	32
COF-LZU1	0.46	5.30	11.5	33
TpPa1	0.48	2.45	5.10	33
Micro-COF-1	0.59	2.9	4.91	34
Micro-COF-2	0.71	3.5	4.93	34
Meso-COF-3	0.84	4.0	4.76	34
Meso-COF-3	1.01	3.3	3.26	34
iCOF-AB-33	1.39	9.00	6.47	35
**Activated Carbon**				
Uassis-PC800	1.67	2.25	1.35	36
KOH-AC	1.15	3.76	3.26	37
AC	0.74	2.42	3.27	37
AC	0.5	0.76	1.52	38
**Polymers**				
PAF-23	0.04	2.71	67.7	39
PAF-24	0.10	2.76	27.6	39
PAF-25	0.20	2.60	13.0	39
AzoPPN	0.68	2.90	4.26	40
PSIF-5	1.41	4.85	3.44	41
SCMP-11	0.62	3.45	5.56	42
TTPB	0.13	4.43	31.0	43
TTPPA	0.30	4.90	16.3	44
TatPOP-2	0.18	4.50	25.0	45

4. PXRD patterns

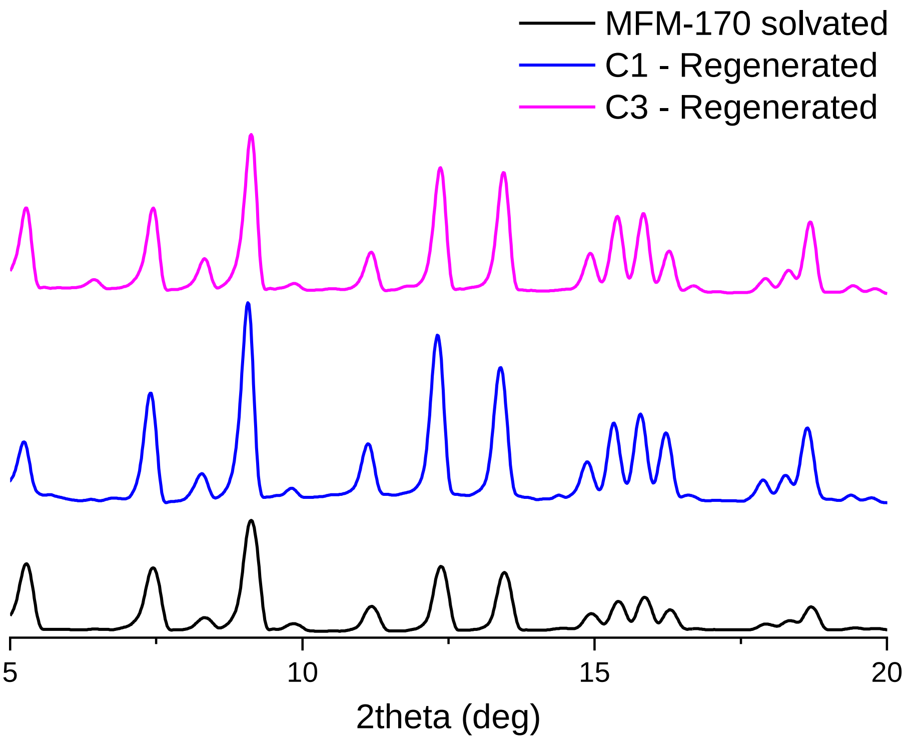

Figure S16. PXRD patterns for MFM-170 and regenerated MFM-170 after I_2 adsorption.

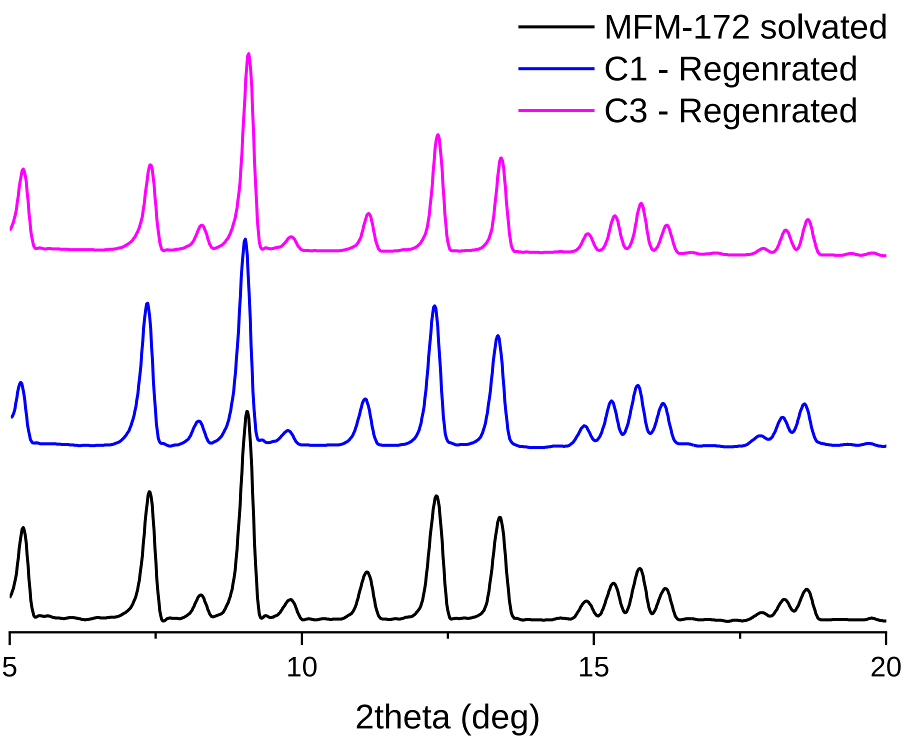

Figure S17. PXRD patterns for MFM-172 and regenerated MFM-172 after I_2 adsorption

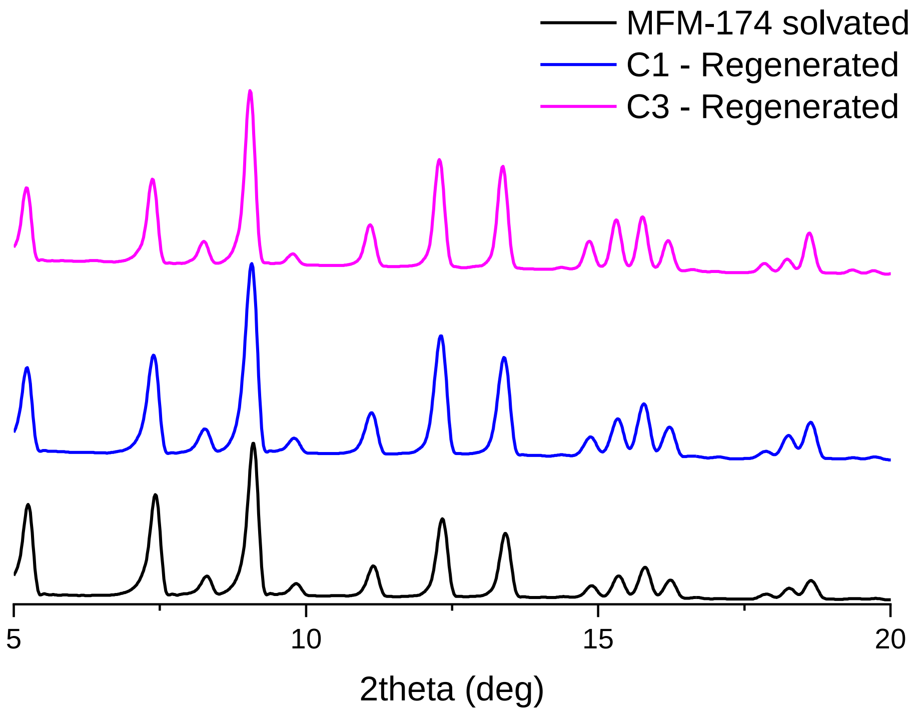

Figure S18. PXRD patterns for MFM-174 and regenerated MFM-174 after I_2 adsorption

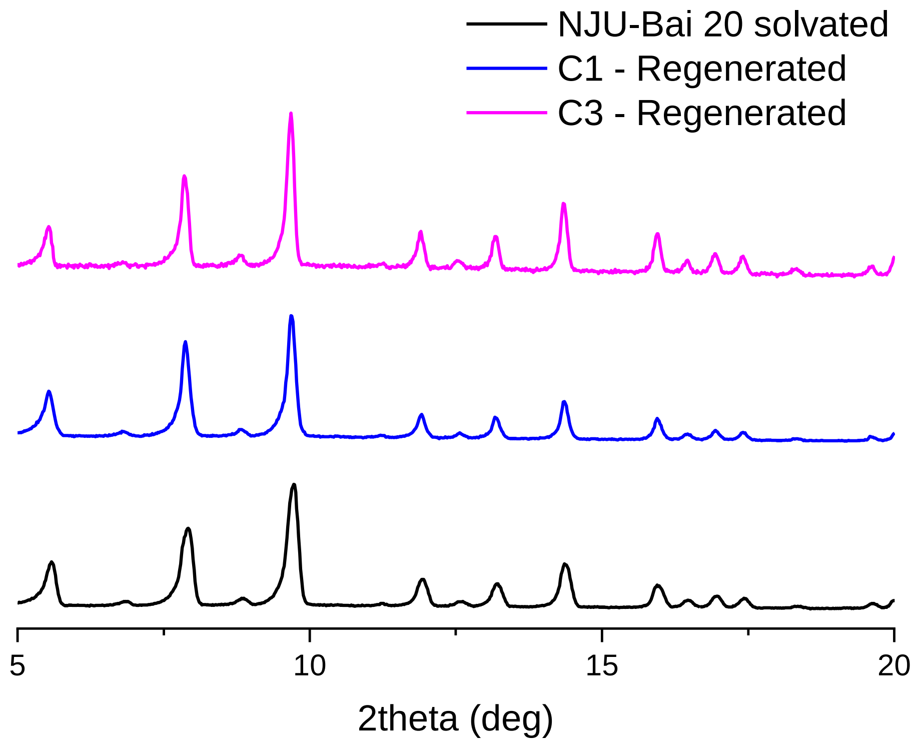

Figure S19. PXRD patterns for NJU-Bai20 and regenerated NJU-Bai20 after I_2 adsorption

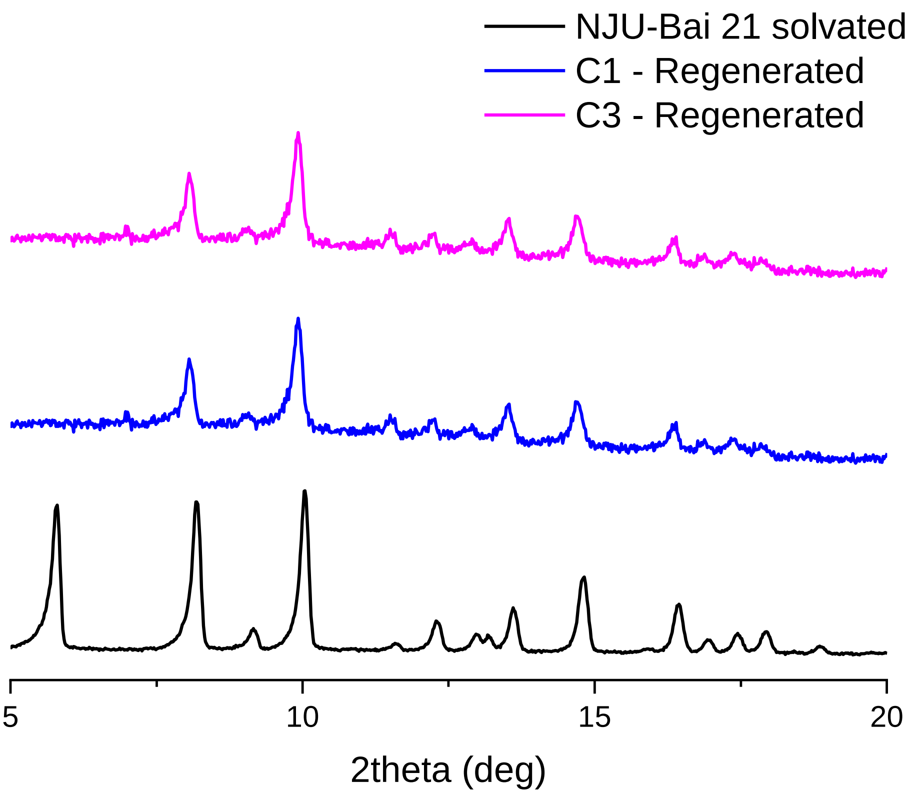

Figure S20. PXRD patterns for NJU-Bai21 and regenerated NJU-Bai21 after I_2 adsorption

5. SEM-EDX images

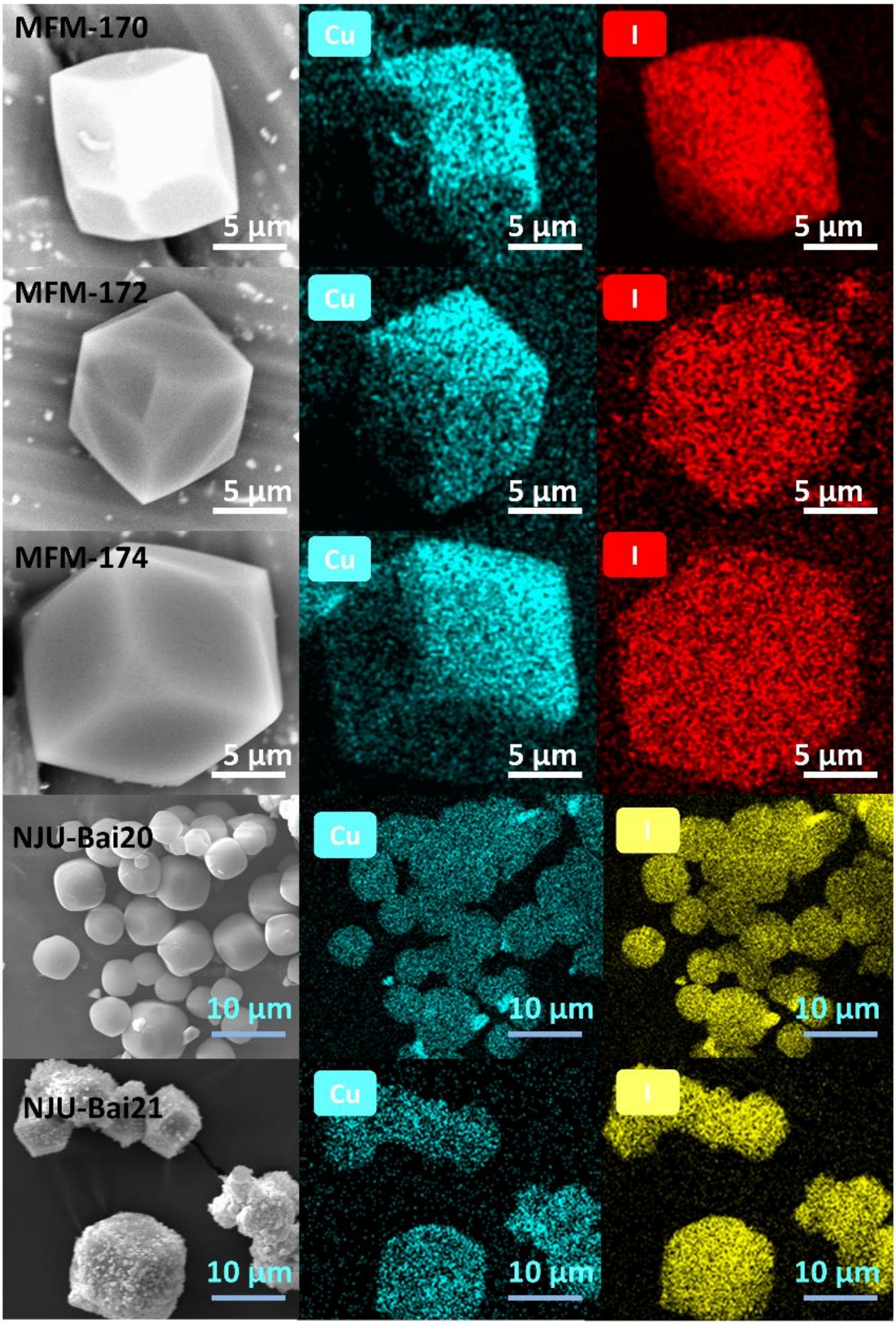

Figure S21. SEM-EDX for I_2-loaded MFM-17x and I_2-loaded NJU-Bai-2x series.

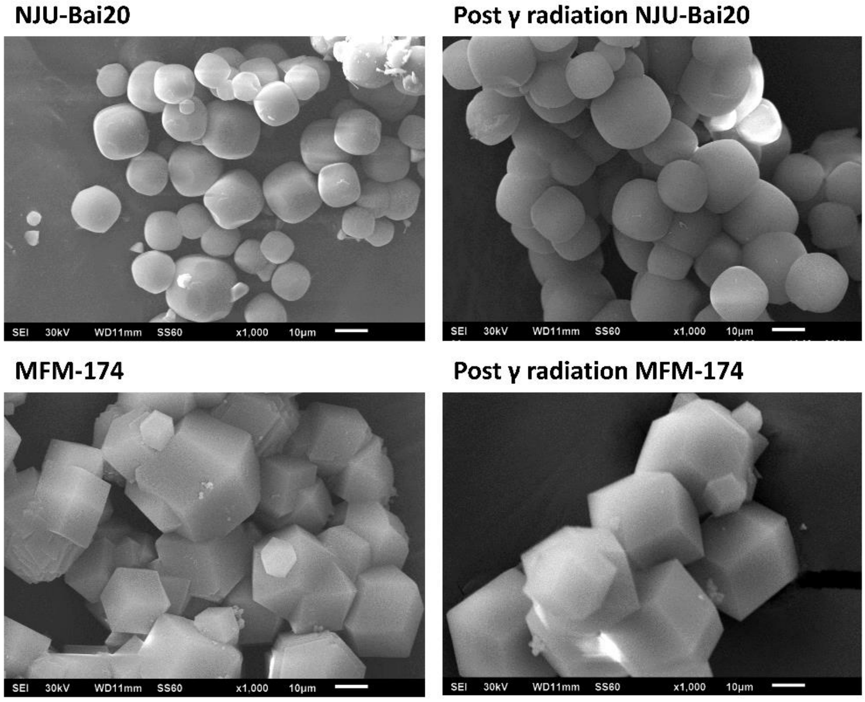

Figure S22. SEM images of fresh MFM-174 and NJU-Bai20 and post γ-irradiation confirming the retention of the morphology.

6. ssNMR spectra

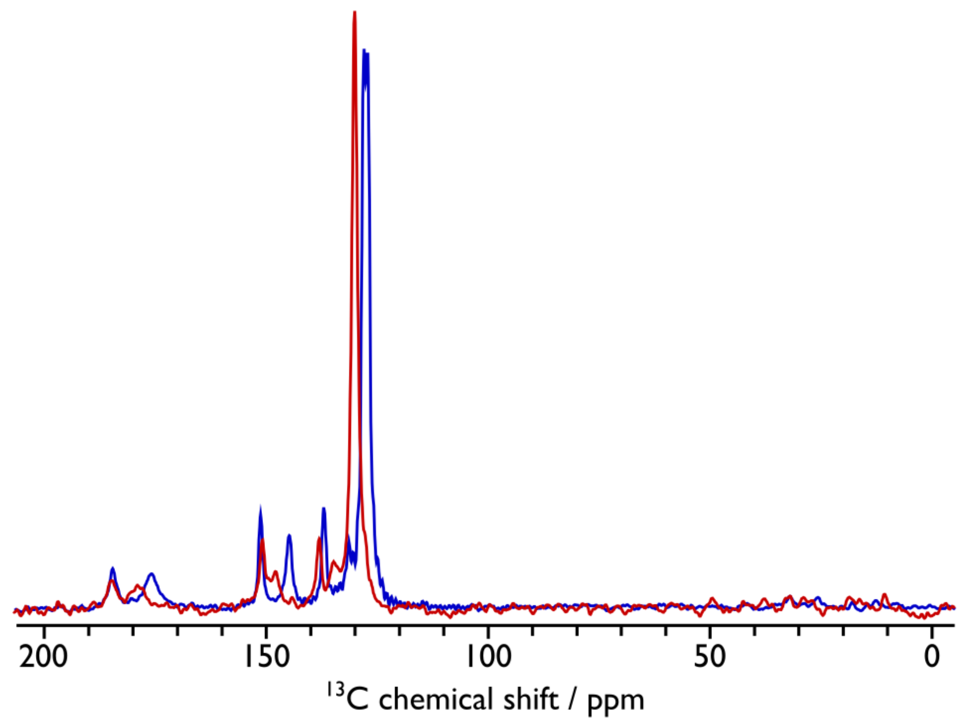

Figure S23. {^{1}H-}^{13}C CPMAS NMR spectra of bare MFM-170 (blue) and I_2@MFM-170 (red).

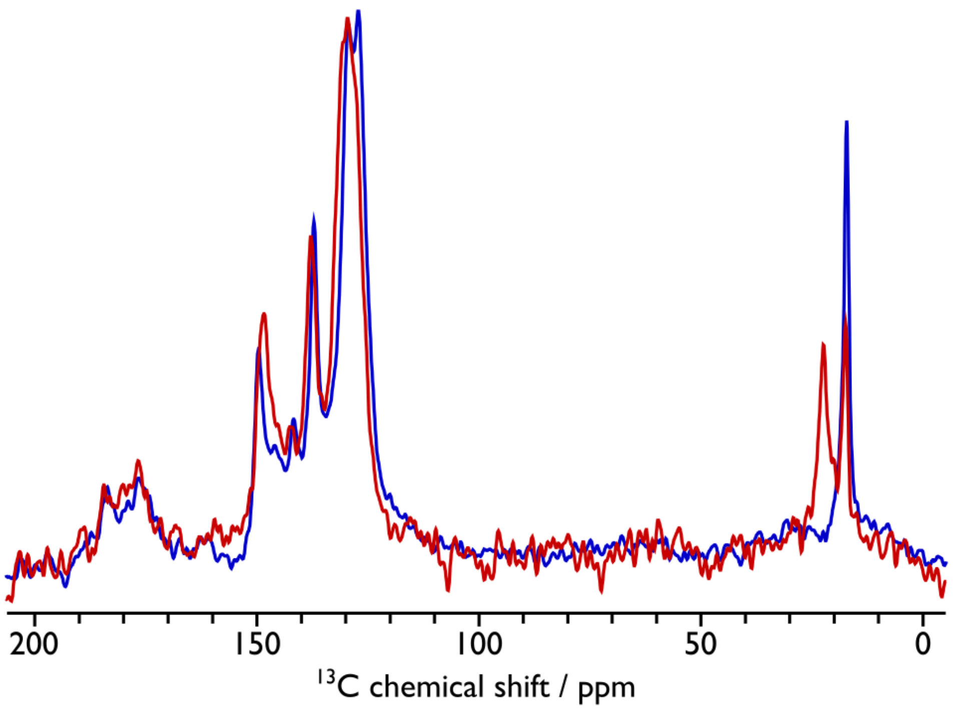

Figure S24. {^{1}H-}^{13}C CPMAS NMR spectra of bare MFM-172 (blue) and I_2@MFM-172 (red).

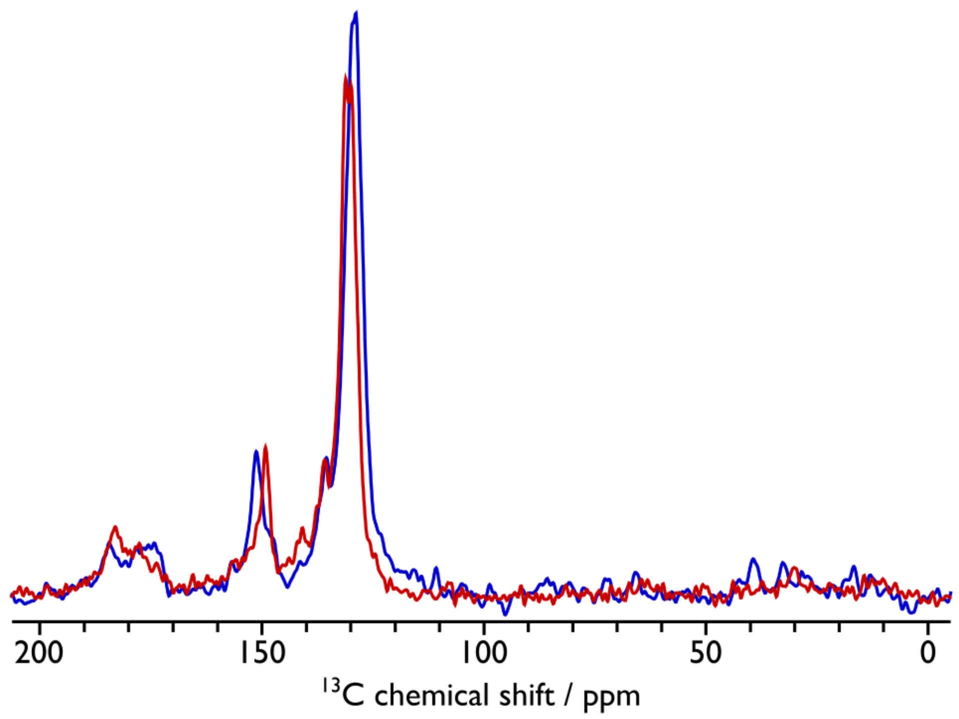

Figure S25. {^{1}H-}^{13}C CPMAS NMR spectra of bare MFM-174 (blue) and I_2@MFM-174 (red).

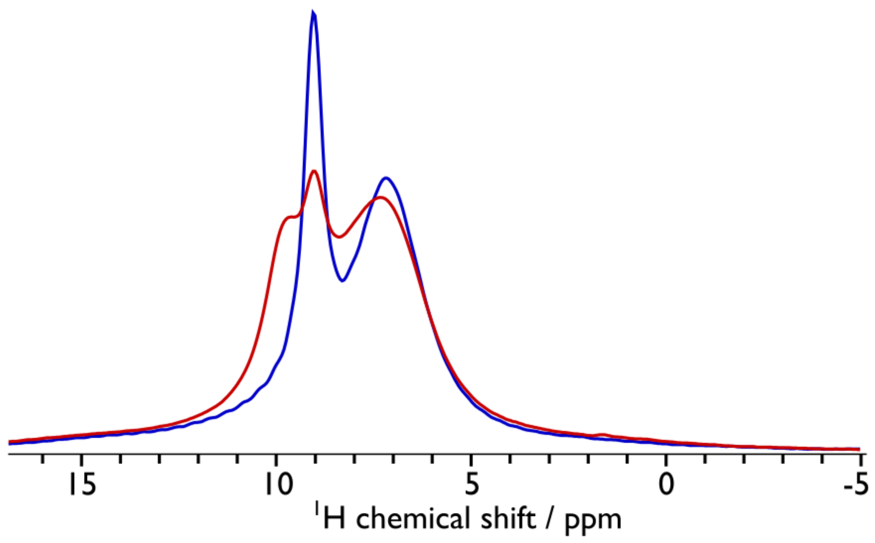

Figure S26. ^{1}H MAS NMR spectra of bare NJU-Bai 20 (blue) and I_2@NJU-Bai 20 (red).

7. X-ray photoelectron spectra (XPS)

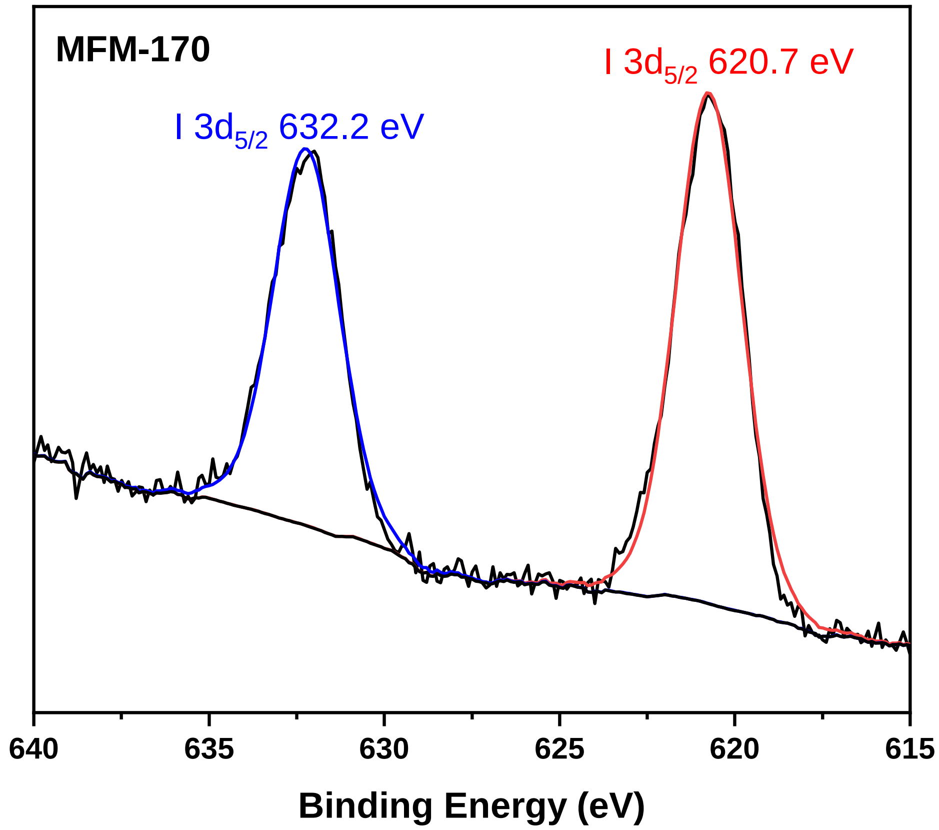

Figure S27. XPS spectra of I_2@MFM-170.

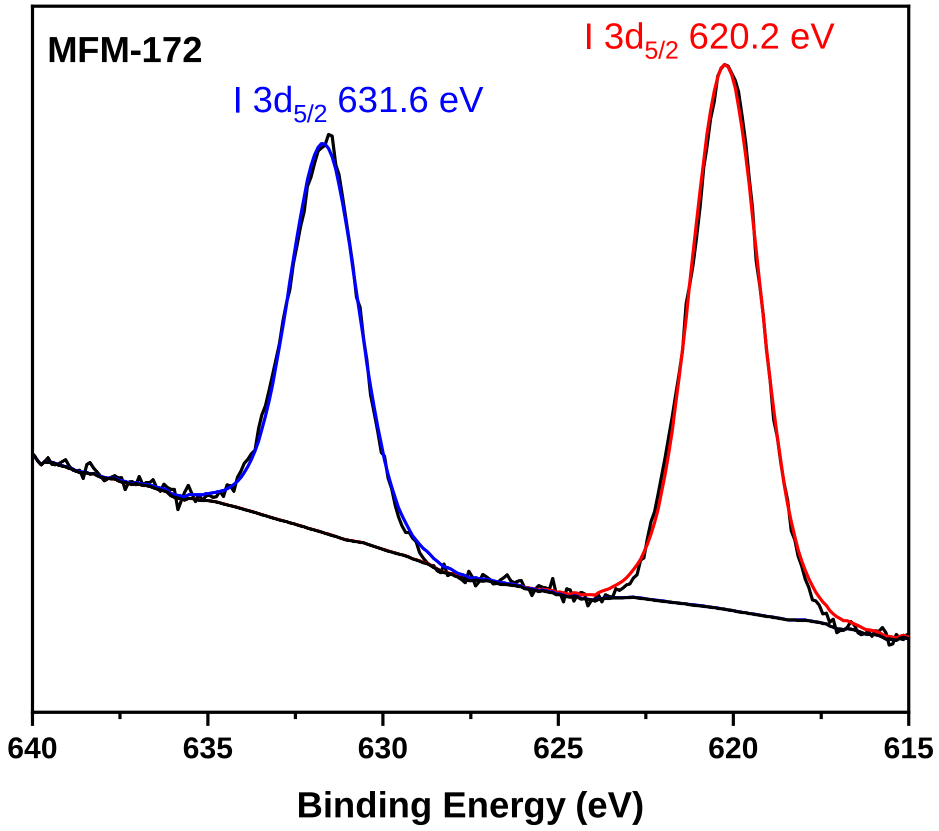

Figure S28. XPS spectra of I_2@MFM-172.

8. Infrared spectra (IR)

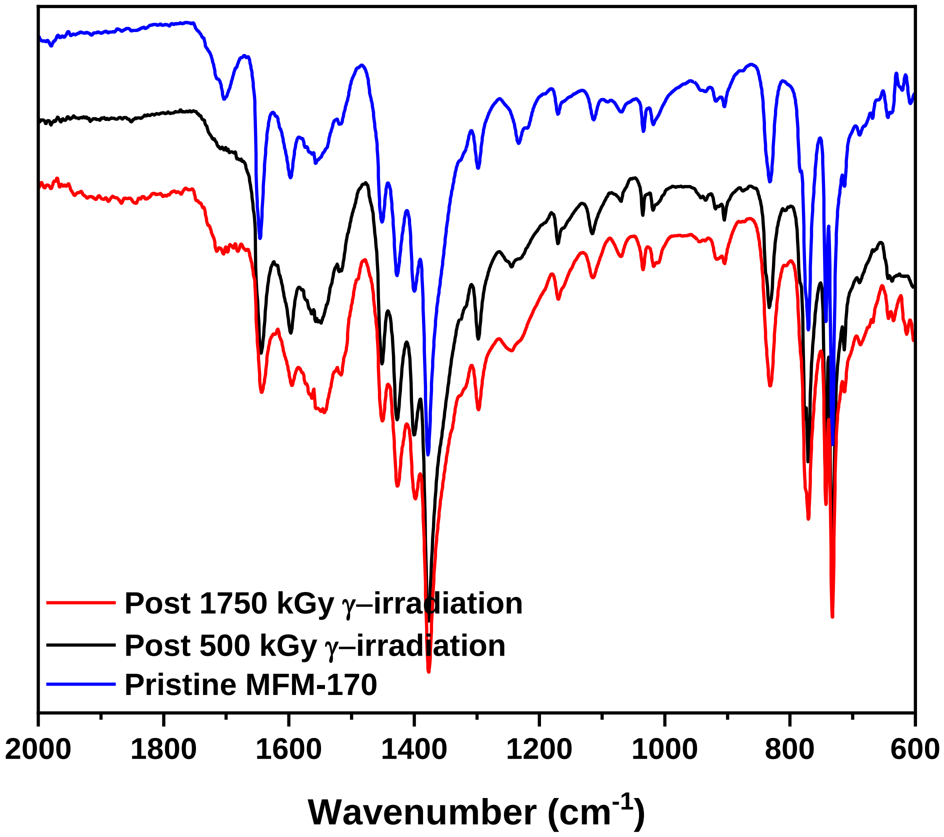

Figure S29. Infrared spectra of MFM-170 and post γ-irradiation of MFM-170.

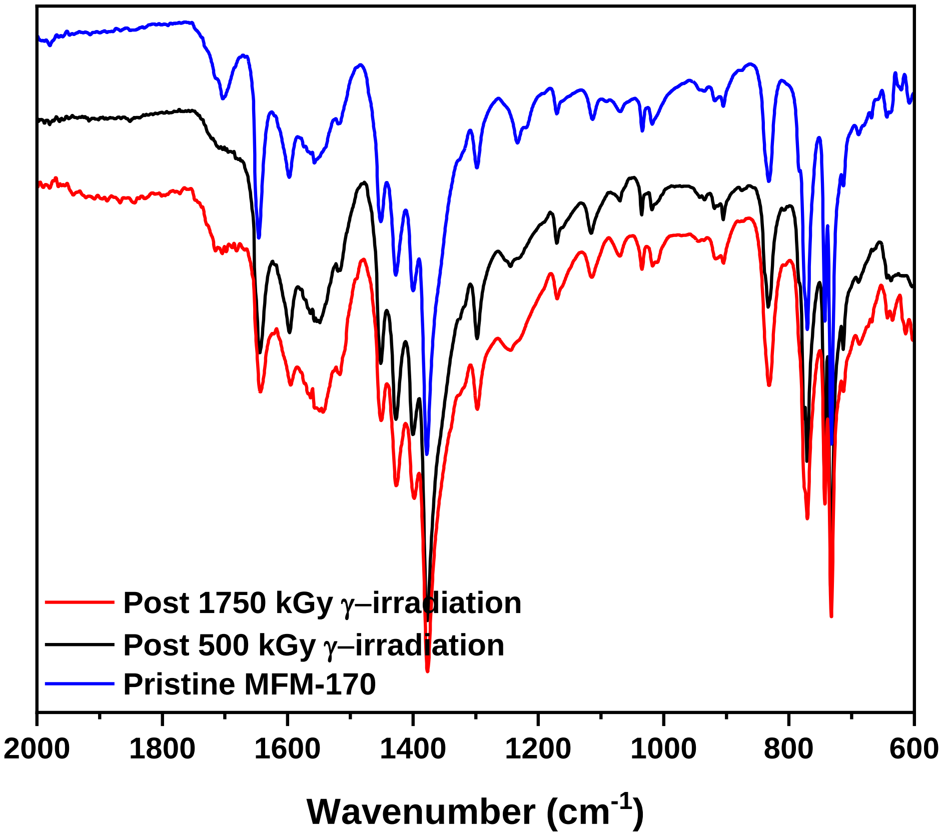

Figure S30. Infrared spectra of MFM-174 and post γ-irradiation of MFM-174.

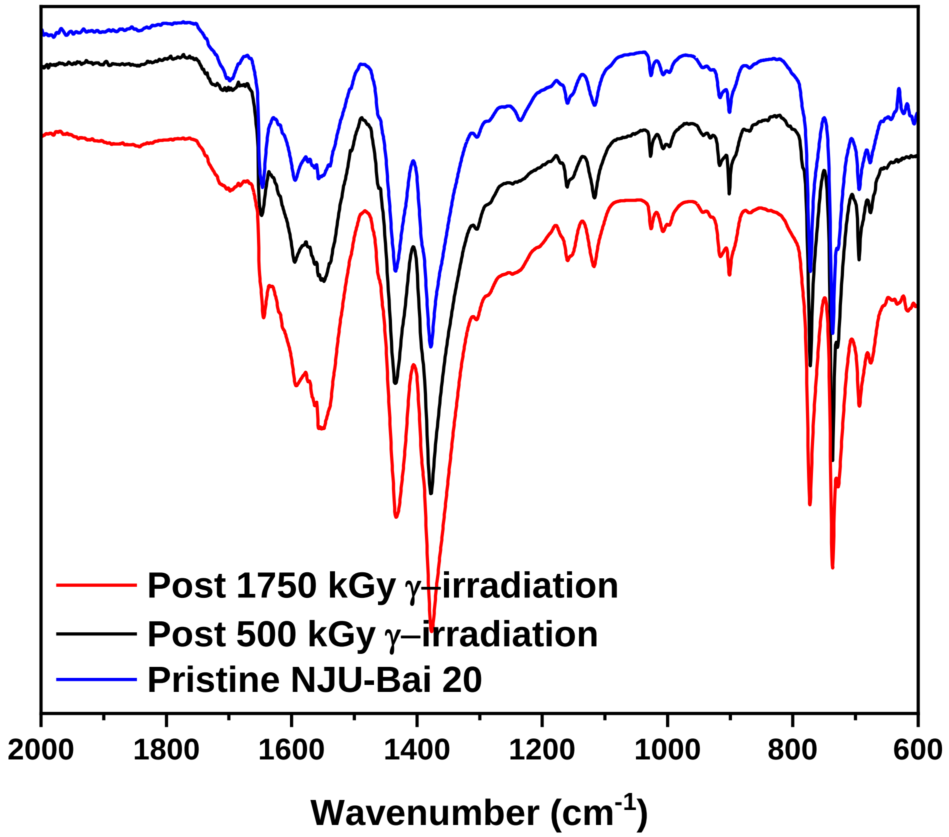

Figure S31. Infrared spectra of NJU-Bai 20 and post γ-irradiation of NJU-Bai 20.

9. Raman spectra

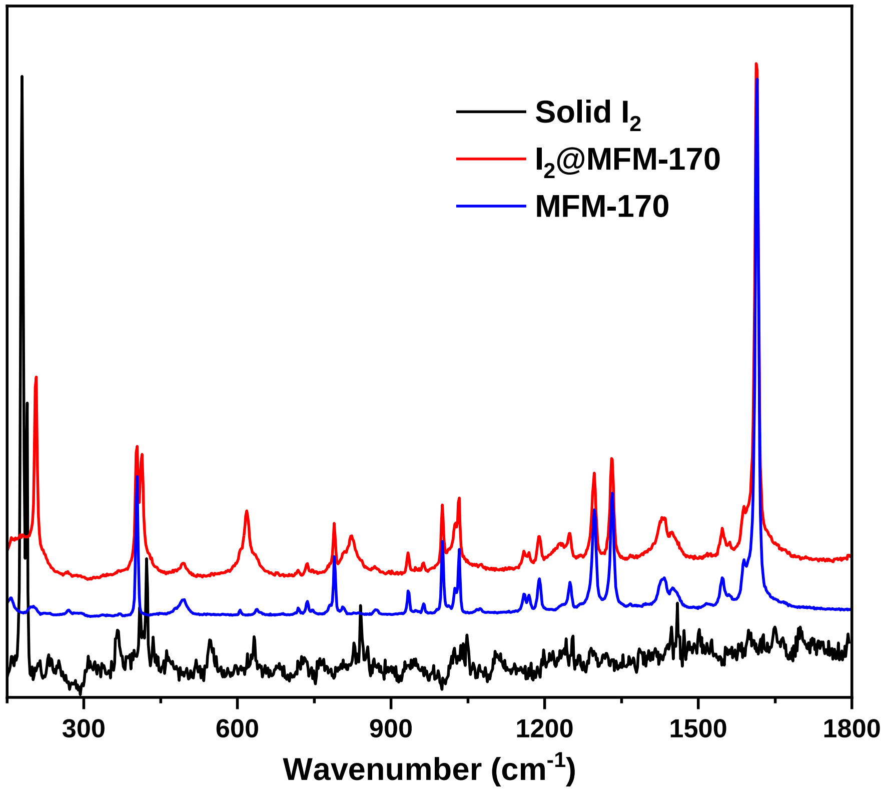

Figure S32. Raman spectra of solid I_2, MFM-170 and I_2@MFM-170.

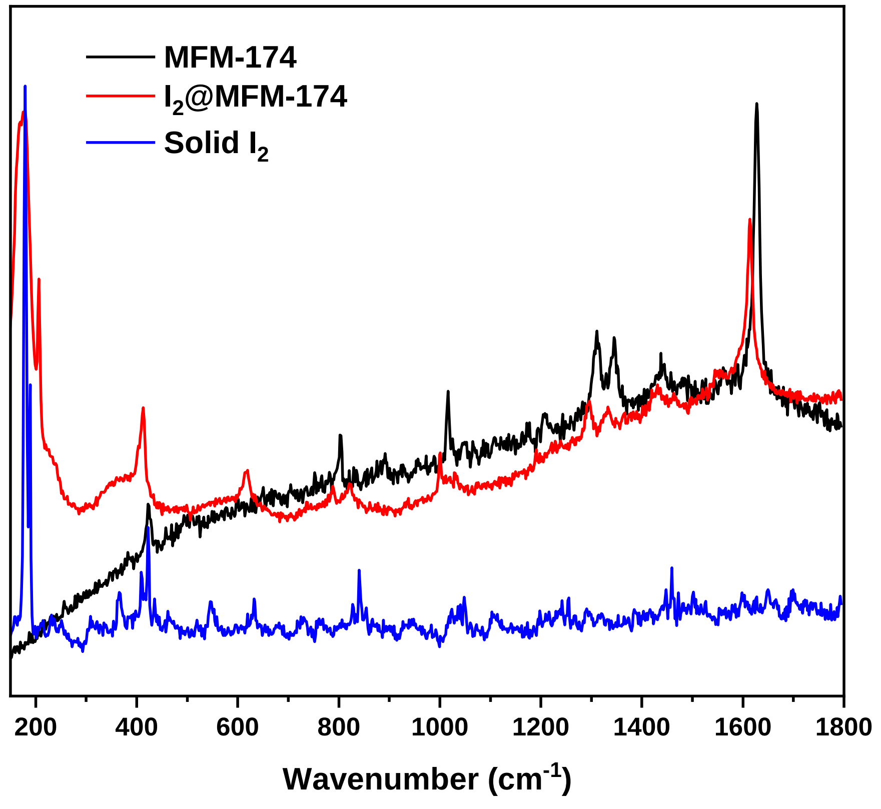

Figure S33. Raman spectra of solid I_2, MFM-174 and I_2@MFM-174.

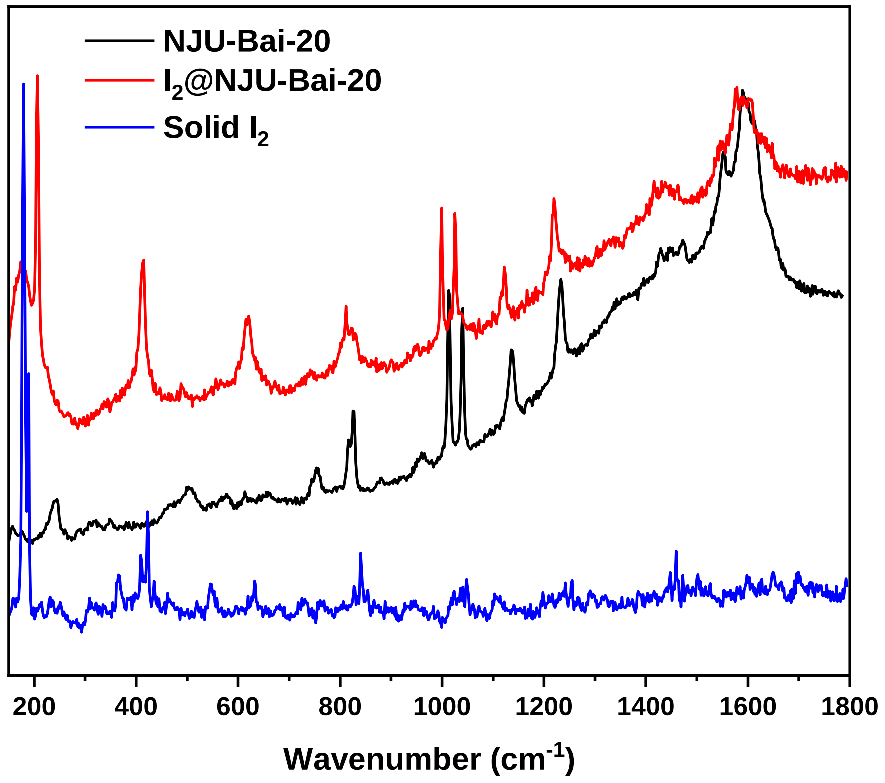

Figure S34. Raman spectra of solid I_2, NJU-Bai20 and I_2@NJU-Bai20.

10. N_2 adsorption isotherms

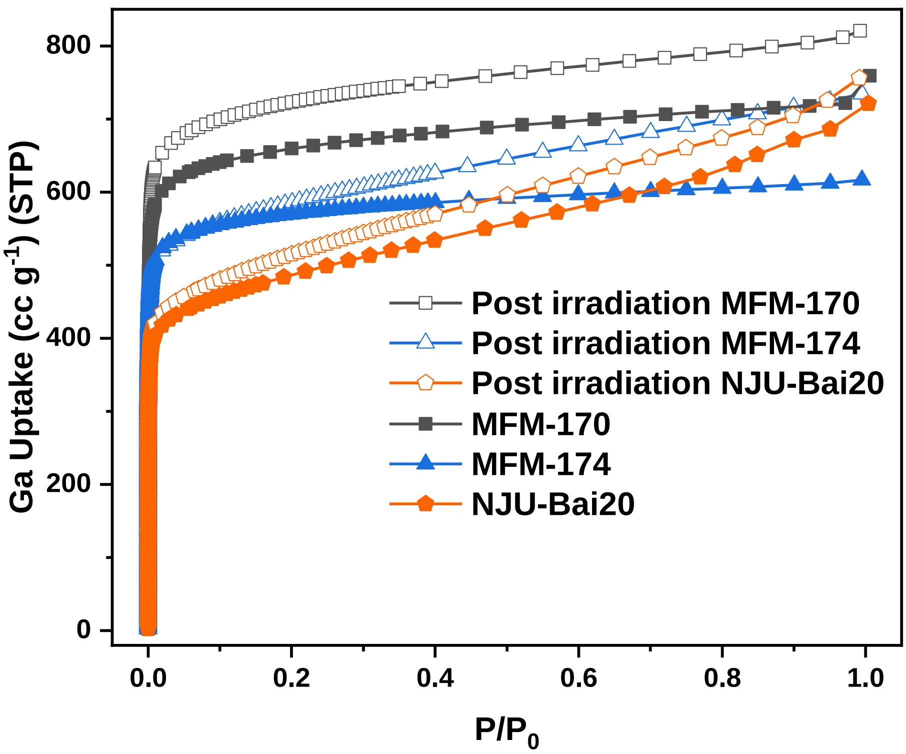

Figure S35. N_2 adsorption isotherms of MFM-170, MFM-174 and NJU-Bai20 and post γ-irradiation at 77 K confirming the retention of the porosity.

11. References

1. Smith, G., Eyley, J., Han, X., Zhang, X., Li, J., Jacques, N., Godfrey, H., Argent, S., McPherson, L., Teat, S., Cheng, Y., Frogley, M., Cinque, G., Day, S., Tang, C., Easun, T., Rudic, S., Ramirez-Cuesta, A., Yang, S. & Schröder, M. Reversible coordinative binding and separation of sulfur dioxide in a robust metal-organic framework with open copper sites. *Nat. Mater.* **2019**, *18*, 1358-1365.
2. Zhang, P., Li, B., Zhao, Y., Meng, X. & Zhang, T. A novel (3, 36)-connected and self-interpenetrated metal-organic framework with high thermal stability and gas-sorption capabilities. *Chem. Commun.* **2011**, *47*, 7722-7724.
3. Lu, Z., Bai, J., Hang, C., Meng, F., Liu, W., Pan, Y. & You, X. The utilization of amide groups to expand and functionalize metal-organic frameworks simultaneously. *Chem. Eur. J.* **2016**, *22*, 6277-6285.
4. Fung, D. M., Khitrin, A. K. & Ermolaev, K. An improved broadband decoupling sequence for liquid crystals and solids. *J. Magn. Reson.* **2000**, *142*, 97-101.
5. Nowell, H., Barnett, S., Christensen, K., Teat, S. & Allan, D. I19, the small-molecule single-crystal diffraction beamline at Diamond Light Source. *J. Synchrotron Rad.* **2012**, *19*, 435-441.
6. CrysAlisPRO, Oxford Diffractions /Agilent Technologies UK Ltd, Tarnton, England.
7. Winter, G., Waterman, D., Parkhurst, J., Brewster, A., Gildea, R., Gerstel, M., Fuentes-Montero, L., Vollmar, M., Michels-Clark, T., Young, I., Sauter, N. & Evams, G. DIALS: implementation and evaluation of a new integration package. *Acta Crystallogr D Struct Biol,* **2018**, *74*, 85-97.
8. Sheldrick, M. G. SHELXT - Integrated space-group and crystal-structure determination. *Acta. Cryst.* A **2015,** *71*, 3-8.
9. Sheldrick, M. G. Crystal structure refinement with SHELXL. *Acta. Cryst.* C **2015**, *71*, 3-8.
10. Dolomanov, V. O., Bourhis, J. L., Gildea, J. R., Howard, J. A. K. & Puschmann, H. OLEX2: a complete structure solution, refinement and analysis program. *J. Appl. Cryst*. **2009**, *42*, 339-341.
11. Sava, D., Chapman, K., Rodriguez, M., Greathouse, J., Crozier, P., Zhao, H., Chupas, P. & Nenoff, T. Competitive I_2 sorption by Cu-BTC from humid gas streams. *Chem. Mater.* **2013**, *25*, 2591–2596.
12. Zhu, X., Zhao, W., Wang, C., Li, A. & Dong, B. Micro-Cu_4I_4-MOF: Reversible iodine adsorption and catalytic properties for tandem reaction of Friedel-Crafts alkylation of indoles with acetals. *Chem. Commun.* **2016**, *52*, 12702–12705.
13. Wang, Z., Zhang, Y., Liu, T., Kurmoo, M. & Cao, S. [$Fe_3(HCOO)_6$]: A permanent porous diamond framework displaying H_2/N_2 adsorption, guest inclusion, and guest-dependent magnetism. *Adv. Funct. Mater*. **2007**, *17*, 1523–1536.
14. Banerjee, D., Chen, X., Lobanov, S., Plonka, A., Chan, X., Daly, J., Kim, T., Thallapally, P. & Parise, J. Iodine Adsorption in metal organic frameworks in the presence of humidity. *ACS Appl. Mater. Interfaces* **2018**, *10*, 10622–10626.
15. Safarifard, V. & Morsali, A. Influence of an amine group on the highly efficient reversible adsorption of iodine in two novel isoreticular interpenetrated pillared-layer microporous metal-organic frameworks. *CrystEngComm* **2014**, *16*, 8660–8663.
16. Zeng, H., Wang, Q., Tan, Y., Hu, S., Zhao, H., Long, L. & Kurmoo, M. Rigid pillars and double walls in a porous metal-organic framework: Single-crystal to single-crystal, controlled uptake and release of iodine and electrical conductivity. *J. Am. Chem. Soc.* **2010**, *132*, 2561–2563.
17. Arici, M., Yeşilel, O. Z., Taş, M. & Demiral, H. Effect of solvent molecule in pore for flexible porous coordination polymer upon gas adsorption and iodine encapsulation. *Inorg. Chem.* **2015**, *54*, 11283–11291.

18. Marshall, J., Griffin, L., Wilson, C. & Forgan, S. Stereoselective halogenation of integral unsaturated C-C bonds in chemically and mechanically robust Zr and Hf MOFs. *Chem. A Eur. J.* **2016**, *22*, 4870–4877.
19. Zhang, X., Silva, I., Godfrey, H., Callear, S., Sapchenko, S., Cheng, Y., Vitorica-Yrezabal, I., Frogley, M., Cinque, G., Tang, C., Giacobbe, C., Dejoie, C., Rudic, S., Ramirez-Cuesta, A., Denecke, M., Yang, S. & Schröder, M. Confinement of iodine molecules into triple-helical chains within robust metal-organic frameworks. *J. Am. Chem. Soc.* **2017**, *139*, 16289–16296.
20. Guo, B., Li, F., Wang, C., Zhang, L. & Sun, D. A rare (3,12)-connected zirconium metal-organic framework with efficient iodine adsorption capacity and pH sensing. *J. Mater. Chem.* A **2019,** *7*, 13173–13179.
21. He, T., Xu, X., Ni, B., Lin, H., Li, C., Hu, W. & Wang, X. Metal–organic framework based microcapsules. *Angew. Chem. Int. Ed.* **2018**, *57*, 10148–10152.
22. Valizadeh, B., Nguyen, N., Smit, B. & Stylianou, C. Porous metal–organic framework@polymer beads for iodine capture and recovery using a gas-sparged column. *Adv. Funct. Mater.* **2018**, *28*, 1–6.
23. Munn, A., Millange, F., Frigoli, M., Guillou, N., Falaise, C., Stevenson, V., Volkringer, C., Loiseau, T., Cibin, G. & Walton, R.Iodine sequestration by thiol-modified MIL-53(Al). *CrystEngComm* **2016**, *18,* 8108–8114.
24. Yao, R. X., Cui, X., Jia, X. X., Zhang, F. Q. & Zhang, X. M. A luminescent zinc(II) metal-organic framework (MOF) with conjugated π -electron ligand for high iodine capture and nitro-explosive detection. *Inorg. Chem.* **2016**, *55*, 9270–9275.
25. Chen, P., He, X., Pang, M., Dong, X., Zhao, S. Zhang, W. Iodine capture using Zr-based metal-organic frameworks (Zr-MOFs): Adsorption performance and mechanism. *ACS Appl. Mater. Interfaces* **2020**, *12,* 20429–20439.
26. Tang, Y., Huang, H., Li, J., Xue, W. & Zhong, C. IL-induced formation of dynamic complex iodide anions in IL@MOF composites for efficient iodine capture. *J. Mater. Chem.* A **2019**, *7*, 18324–18329.
27. Maddock, J., Kang, X., Liu, L., Han, B., Yang, S. & Schröder, M. The impact of structural defects on iodine adsorption in UiO-66. *Chemistry* **2021**, *3*, 525-531.
28. Guo, X., Li, Y., Zhang, M., Cao, K., Tian, Y., Qi, Y., Li, S., Li, K., Yu, X. & Ma, L. Colyliform crystalline 2D covalent organic frameworks (COFs) with quasi-3D topologies for rapid I_2 adsorption. *Angew. Chem. Int. Ed.* **2020**, *59*, 22697–22705.
29. Wang, P., Xu, Q., Li, Z., Jiang, W., Jiang, Q. & Jiang, D. Exceptional iodine capture in 2D covalent organic frameworks. *Adv. Mat.* **2018**, *30*, 1801991.
30. Sun, F., Yin, Z., Wang, Q., Sun, D., Zeng, M. & Kurmoo, M. Tandem postsynthetic modification of a metal–organic framework by thermal elimination and subsequent bromination: effects on absorption properties and photoluminescence. *Angew. Chem., Int. Ed.* **2013**, *52*, 4538–4543.
31. Guo, X., Tian, Y., Zhang, M., Li, Y., Wen, R., Li, X., Li, X., Xue, Y., Ma, L., Xia, C. & S. Li, Mechanistic insight into hydrogen-bond-controlled crystallinity and adsorption property of covalent organic frameworks from flexible building blocks. *Chem. Mater.* **2018**, *30*, 2299–2308.
32. Yin, Z., Xu, S., Zhan, T., Qi, Q., Wu, Z. & Zhao, X. Ultrahigh volatile iodine uptake by hollow microspheres formed from a heteropore covalent organic framework. *ChemComm.* **2017**, *53*, 7266–7269.
33. Das, G., Skorjanc, T., Sharma, S., Gandara, F., Lusi, M., Shankar, M., Vimala, S., Prasad, K., Raya, J., Han, D., Jagannathan, R., Olsen, J. & Trabolsi, A. Viologen-based conjugated covalent organic networks *via* zincke reaction. *J. Am. Chem. Soc.* **2017**, *139*, 9558–9565.

34. Weber, J., Su, Q., Antonietti, M. & Thomas, A. Exploring polymers of intrinsic microporosity-microporous, soluble polyamide and polyimide. *Macromol. Rapid Commun*. **2007**, *28,* 1871–1876.
35. Xie, Y., Pan, T., Lei, Q,, Chen, C., Dong, X., Yuan, Y., Shen, J., Cai, Y., Zhou, C., Pinnau, I. & Han, Y. Ionic functionalization of multivariate covalent organic frameworks to achieve an exceptionally high iodine-capture capacity. *Angew. Chem., Int. Ed.* **2021**, *60*, 22432-22440.
36. Xiao, K., Liu, H., Li, Y., Yang, G., Wang, Y. & Yao, H. Excellent performance of porous carbon from urea-assisted hydrochar of orange peel for toluene and iodine adsorption. *Chem. Eng. J.* **2020**, *382*, 122997.
37. Sun, H., La, P., Zhu, Z., Liang, W., Yang, B. & Li, A. Capture and reversible storage of volatile iodine by porous carbon with high capacity. *J. Mater. Sci.* **2015**, *50*, 7326–7332.
38. Huang, P., Cheng, H. & Lin, S. Adsorption of carbon dioxide onto activated carbon prepared from coconut shells *J. Chem.* **2015**, 106590.
39. Yan, Z., Yuan, Y., Tian, Y., Zhang, D. & Zhu, G. Highly efficient enrichment of volatile iodine by charged porous aromatic frameworks with three sorption sites. *Angew. Chem. Int. Ed.* **2015**, *54*, 12733–12737.
40. Shi, B., Pei, R., Chen, A., Wang, C., Ma, Y. & Yin, Z. Efficient iodine capture by metal–organic cubes based on hexanuclear vanadium cluster. *Inorg. Chem. Commun.* **2019**, *102*, 147–151.
41. He, T., Xu, X., Ni, B., Lin, H., Li, C., Hu, W. & Wang, X. Metal–organic framework based microcapsules. *Angew. Chem. Int. Ed.* **2018**, *57*, 10148–10152.
42. Ren, F., Zhu, Z., Qian, X., Liang, W., Mu, P., Sun, H., Liu, J. & Li, A. Novel thiophene-bearing conjugated microporous polymer honeycomb-like porous spheres with ultrahigh iodine uptake. *ChemComm*. **2016**, *52*, 9797–9800.
43. Geng, T., Zhu, Z., Zhang, W. & Wang, Y. A nitrogen-rich fluorescent conjugated microporous polymer with triazine and triphenylamine units for high iodine capture and nitro aromatic compound detection. *J. Mater. Chem. A* **2017**, *5*, 7612–7617.
44. Geng, T., Ye, S., Zhu, Z. & Zhang, W. Triazine-based conjugated microporous polymers with N,N,N',N'-tetraphenyl-1,4-phenylenediamine, 1,3,5-tris(diphenylamino)benzene and 1,3,5-tris[(3-methylphenyl)-phenylamino]benzene as the core for high iodine capture and fluorescence sensing of o-nitrophenol. *J. Mater. Chem. A* **2018**, *6*, 2808–2816.
45. Valizadeh, B., Nguyen, T., Smit, B. & Stylianou, K. Porous metal–organic framework@polymer beads for iodine capture and recovery using a gas-sparged column. *Adv. Funct. Mater.* **2018**, *28*, 1–6.
